# Essential Roles for Soluble Virion-Associated Heparan Sulfonated Proteoglycans and Growth Factors in Human Papillomavirus Infections

**DOI:** 10.1371/journal.ppat.1002519

**Published:** 2012-02-09

**Authors:** Zurab Surviladze, Agnieszka Dziduszko, Michelle A. Ozbun

**Affiliations:** Department of Molecular Genetics & Microbiology, University of New Mexico School of Medicine, Albuquerque, New Mexico, United States of America; University of Michigan, United States of America

## Abstract

A subset of human papillomavirus (HPV) infections is causally related to the development of human epithelial tumors and cancers. Like a number of pathogens, HPV entry into target cells is initiated by first binding to heparan sulfonated proteoglycan (HSPG) cell surface attachment factors. The virus must then move to distinct secondary receptors, which are responsible for particle internalization. Despite intensive investigation, the mechanism of HPV movement to and the nature of the secondary receptors have been unclear. We report that HPV16 particles are not liberated from bound HSPG attachment factors by dissociation, but rather are released by a process previously unreported for pathogen-host cell interactions. Virus particles reside in infectious soluble high molecular weight complexes with HSPG, including syndecan-1 and bioactive compounds, like growth factors. Matrix mellatoproteinase inhibitors that block HSPG and virus release from cells interfere with virus infection. Employing a co-culture assay, we demonstrate HPV associated with soluble HSPG-growth factor complexes can infect cells lacking HSPG. Interaction of HPV-HSPG-growth factor complexes with growth factor receptors leads to rapid activation of signaling pathways important for infection, whereas a variety of growth factor receptor inhibitors impede virus-induced signaling and infection. Depletion of syndecan-1 or epidermal growth factor and removal of serum factors reduce infection, while replenishment of growth factors restores infection. Our findings support an infection model whereby HPV usurps normal host mechanisms for presenting growth factors to cells *via* soluble HSPG complexes as a novel method for interacting with entry receptors independent of direct virus-cell receptor interactions.

## Author Summary

A subset of the >120 different types of human papillomaviruses (HPVs) are the most common cause of sexually transmitted infections. Certain HPVs are also associated with approximately 5% of all cancers worldwide. Like many pathogens, HPVs bind first to heparan sulfate proteoglycans (HSPGs) on cells before moving to more specific uptake receptors. However, relatively little is known about the mechanism(s) that triggers the translocation of HPV from HSPGs to the receptors that facilitate entry. As obligate parasites, viruses have evolved numerous means to hijack host cell functions to cause infection. We report two novel mechanisms of pathogen-host interactions. First, bound HPV particles are liberated from cells in an active complex with HSPGs and growth factors rather than dissociating from the sugars to engage secondary receptors. Second, HPV uses the specificity of the associated growth factors to bridge to their cognate receptors as opposed to direct binding to a cell internalization receptor. Signals transduced during these interactions are important for HPV infection. Our study provides new insights into the transmission of a significant viral pathogen and reveals novel means whereby microbes may repurpose normal cell functions during infection of their hosts. Likewise, this work uncovers new targets for HPV prophylaxis.

## Introduction

Human papillomaviruses (HPVs) are small, DNA-containing viruses that infect mucosal and cutaneous epithelium to cause benign and malignant tumors, including many anogenital, oropharyngeal and some skin cancers [1], [2]. HPVs demonstrate remarkable host restrictions and have strict tropism for stratifying squamous epithelium. HPV virions consist of 360 copies of the L1 capsid protein, 12–72 copies of the L2 protein and the circular viral genome (≈8 kb) condensed by cellular histones. Like a number of other pathogens, HPV entry into target cells is a multistep process initiated by binding to cell surface attachment factors, the most common of which are glycosaminoglycan chains, especially heparan sulfate in proteoglycans (HSPGs) [3], [4]. Binding to these negatively charged polysaccharides is usually electrostatic and relatively nonspecific. Many microbes like HPVs must transfer from HSPG to a distinct secondary receptor responsible for active pathogen internalization [5]. For HPVs this entry receptor has been elusive. Despite intensive investigation, the mechanism of HPV movement from primary HSPG attachment receptors to secondary high-affinity receptors has been unclear.

Several studies suggest a role for HPV L2 protein in facilitating infection *via* interaction with a secondary receptor (reviewed in ref. [6]). In this model, initial virus attachment to HSPG causes a conformational change in L1 that facilitates a critical proteolytic cleavage of L2 by furin, a proprotein convertase [6]–[8]. L2 cleavage is thought to expose the L2 binding site for the secondary cell receptor, lowering the affinity of L1 for HSPG binding and resulting in transfer to the entry receptor [8]. Many, but not all, of the accumulating experimental data support this attractive hypothesis. Although antibodies raised to L2 can neutralize infection [9] and *in vitro* synthesized L2 peptides and proteins can interact with the cell surface [10], [11], there is no direct evidence that L2 in the context of the virion has a function at the cell plasma membrane. Scatchard plot analyses indicate high affinity binding of HPV33 VLP to HeLa cells, with a K_d_ of ∼85×10^−12^ M [12]. This strong binding affinity of L1 VLP for cells makes it difficult to conceive how cleavage of L2, which is not involved in primary binding, could change the affinity of L1 so dramatically as to cause particle dissociation from the HS chain. Moreover, a recent report shows that heparin binding does not induce obvious conformational changes in the HPV16 capsid structure *in vitro*, except for slight movements of the surface loops and the residues directly involved in oligosaccharide binding [13]. Additional observations that call into question a function for L2 in early entry steps include the fact that L1-only containing virus-like particles (VLP) are capable of normal internalization in cells [14]–[16] and PsVs containing a furin-resistant L2 mutant bind, enter, and uncoat in the endosome [7]. Finally, furin cleaved HPV particles can be rendered non-infectious by heparinase treatment, suggesting that furin does more than simply altering HPV L2 proteins [17]. These various observations illustrate the uncertainty of how HPV particles move from HSPGs to an internalization receptor.

**Figure 1:**
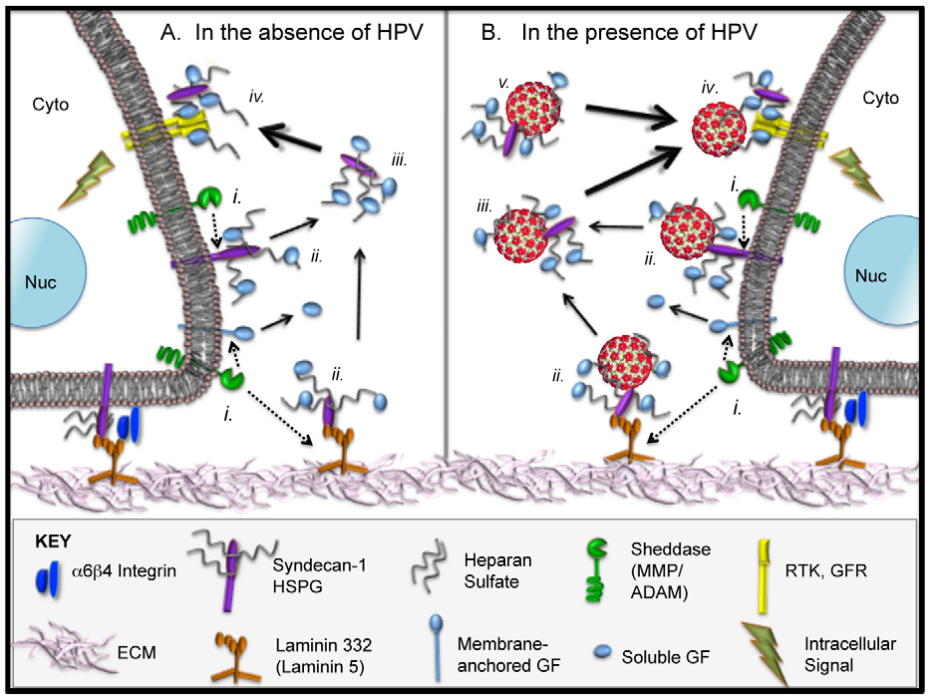
Normal HSPG biology and proposed model for extracellular interactions of HPVs in the context HS-GF complexes. (**A**). Natural processes of HSPG shedding that occur in the absence of HPV. The lower edges of epithelial cell lipid bilayers are depicted interacting with the extracellular matrix (ECM). The ECM consisting of (e.g.) collagens, elastins, fibronectins, laminins is shown in pink. Laminin 332 (formerly laminin 5; orange) interacts with syndecan-1 (purple) and alpha-6 beta-4 integrin (dark blue) on the cell surface to provide cell anchorage to the ECM/basement membrane. Notably, these three molecules have been identified as HPV attachment factors (refs. in text). (***i.***) Sheddases including matrix metalloproteinase (MMP) and ADAM (a disintegrin and metalloproteinase) sheddases (green) normally catalyze the release or “shedding” (dotted arrows) of membrane-bound growth factors (GFs; light blue) and other bioactive molecules, the protein ectodomains of HSPGs like syndecan-1, and ECM residents like laminin 332 [27]. (***ii.***) HSPGs in the plasma membrane and ECM act as local depots for soluble GFs and other bioactive molecules. The HS-GF and bioactive compounds can interact with their cognate receptors laterally, *via* soluble form after release (*iii*), or in the ECM when cells migrate over the HSPG-complexes. (***iii.***) Sheddases including MMPs and heparanases and proteolytic processing of laminin 332 liberate soluble complexes containing GFs and HS/syndecan-1. (***iv.***) Soluble HS-GF complexes bind to GFR/RTK (yellow) and activate intracellular signaling cascades. (**B**). The natural processes of HSPG decoration and release from the cells also occur in the presence of HPV particles (red). The virion image is based on the atomic structure from Modis et al. [109]. By virtue of interaction with HS, HPV can join the complex at each stage where HSPG is involved (***i–iv***). HPV could associate with soluble HS-GF in a naïve infection site or during release from infected cells (***v.***). HPV association with syndecan-1 *via* HSPG and binding of syndecan-1 to laminin 332 and alpha-6 beta-4 integrin are consistent with the fact that HPV particles colocalize and interact with each of these extracellular molecules. The abundance of HSPG in the ECM can explain why HPVs bind at such high levels to the ECM (*ii.*). Cells can pick up HPV-HS-GF complexes in soluble form or by migrating over ECM-bound HPV-HS-GF complexes.

Syndecan-1 is the most abundant HSPG in keratinocytes and is an HPV attachment receptor [18], [19]. Syndecans possess enormous molecular and functional diversity owing to modifications of their HS chains by sulfate groups that vary in sulfation degree, length, charge and sugar composition as well as by covalent attachment of chondroitin sulfate chains [20]. These modifications facilitate the interaction of syndecans and other HSPG with a variety of ligands including growth factors (GFs), cytokines, chemokines, extracellular matrix (ECM) proteins, proteinases and their inhibitors, viruses from such Families as *Retroviridae, Herpesviridae*, *Papillomavirdae* and *Flaviviridae*, as well as several bacterial pathogens [21], [22]. This range of ligand interactions allows HSPG and syndecans to participate in many different cellular activities, including organogenesis, GF and cytokine binding, cellular adhesion, and wound healing. By binding soluble GFs, syndecans are able to concentrate these ligands on or near cells and present them to their high affinity cell surface receptors (depicted in Figure 1A) [23], [24].

A prominent characteristic of syndecans is that their extracellular domains can be cleaved to release intact HS-containing ectodomains decorated with bioactive molecules that act as soluble effectors [25], [26]. All syndecan ectodomains are shed constitutively as a normal part of turnover, but this process is also regulated (e.g., certain GFs accelerate shedding). The enzymes responsible for syndecan shedding are the matrix metalloproteinase peptidases (MMPs) that cleave the syndecan core protein and release the ectodomains (Figure 1A i). MMPs comprise a family of over 25 endopeptidases capable of cleaving all kinds of ECM proteins and cell surface receptors; MMPs also can process a number of bioactive molecules [27]. The HS moieties on syndecans also can be processed by heparinases, which can liberate the HS bound to GFs and bioactive compounds. The many biological functions of shedding syndecans have been summarized in several excellent reviews [21], [23], [28].

Because HPVs are known to interact with HSPGs like syndecan-1 at the cell surface and on the ECM, we investigated whether virus particles bound to these molecules could be released in association with HSPG complexes containing bioactive molecules like GFs. Although syndecan-1 HSPGs associate with a number of soluble biological mediators to present them to their high affinity binding receptors [21], we chose to focus on epidermal growth factor (EGF) and fibroblast growth factor 7 (FGF7, also known as keratinocyte growth factor [KGF]) and their cognate receptors EGFR and KGFR (FGFR2IIIb). These receptors are abundant GFRs on human keratinocytes and play vital roles during wound healing [29], an important mediator of HPV infection of epithelial surfaces [30]. Further, syndecan-1 interactions with EGFR and KGFR ligands are well characterized [21]. We hypothesized that the normal cellular mechanism involving HSPG-GF/bioactive complex release from cells might help explain how HPVs transfer to secondary internalization receptors.

Herein we describe two novel findings with respect to initial HSPG binding by a pathogen and movement to specific uptake receptors on host cells. First, we show that HPV particles bound initially to cell surface HSPGs are released as soluble and infectious high molecular weight (HMW) complexes with HSPGs and GFs. Second, we provide evidence that the HPV-HS-GF complexes activate signaling cascades that are important mediators of HPV infection of human keratinocytes. The data support a model whereby HPVs bind to uptake receptors indirectly *via* a GF bridge between the virus and the cognate GFR.

## Results

### MMPs contribute to cell surface release of HPV, which is important for infection

**Figure 2:**
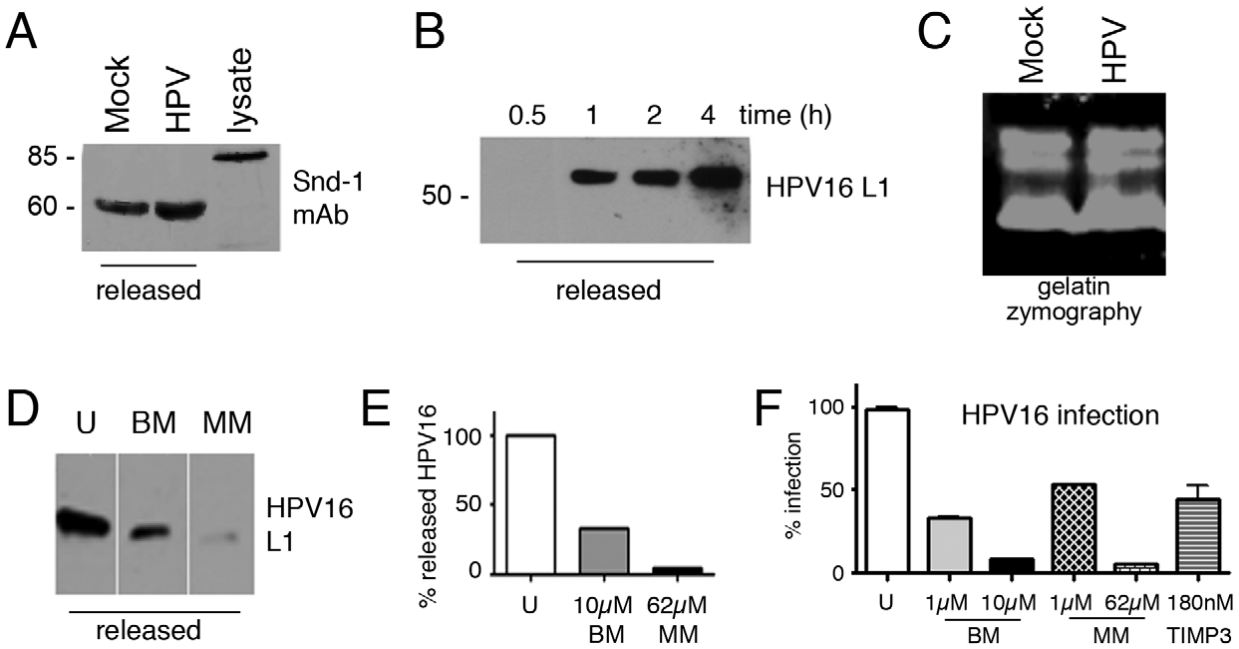
HPV16 and syndecan-1 release from the HaCaT cell plasma membrane is MMP dependent. Immunoblot for syndecan-1 (snd-1) post starvation in SFM and after 6 h at 37°C in Tyrode's Buffer (A); immunoblot for HPV16 L1 released into CM post virus binding for indicated times (B) or 24 h (D). (C) Gelatin zymography showing protease activity present in HaCaT cell CM alone or with binding of HPV16. (D–F) Effect of MMP inhibitors batimastat (BM) or marimastat (MM) at the indicated concentrations on HPV16 release into media as in panel B and densitometric quantification with AlphaEaseFC software (E) and relative infection levels (F). Cells were untreated (U) or pre-treated with the indicated concentrations of BM, MM or TIMP3 for 1 h, then exposed to HPV16 PsV in the presence of inhibitors in CM. Panel D includes lanes spliced together from the same exposure of the same films. (F) Infection was assayed by quantifying luciferase levels at 24 h p.i. Data are represented as mean ± SEM of 3 experiments.

Confocal microscopy and immunoprecipitation (IP) were used to verify HPV16 and HPV31 particles bind to syndecan-1 on HaCaT human keratinocytes (Figure S1). HSPGs including syndecans-1 are actively shed from epithelial cells *via* the activity of a variety of MMP sheddases including, but not limited to, MMP7, MMP9, MT1MMP, ADMTS1, ADAM17, and LasA (Figure 1A*i*) [25], [31]. Therefore, we hypothesized that HSPG-bound HPV particles would be released from cells in complex with HSPGs and syndecan-1. To test this theory we collected media from HaCaT cells growing in complete medium (CM; DMEM containing 10% FCS), including cells exposed to HPV16 PsV, and assayed for released syndecan-1 and HPV. Immunoblot analysis for syndecan-1 showed that CM itself contained substantial levels of syndecan-1 (not shown). This finding complicated the determination of virus binding effect on syndecan-1 release. To avoid the issue of free syndecan-1 in CM, we used serum-free medium (SFM) or Tyrode's buffer solution (see Materials and Methods). Cells starved in SFM were exposed to HPV16 at 4°C and analyzed for syndecan-1 release after 6 h maintenance in Tyrode's buffer at 37°C. Immunoblot showed that cells released a truncated form corresponding to the syndecan-1 ectodomain, whereas the monoclonal antibody detected the SDS-stable dimeric form of syndecan-1 form in cell lysates (Figure 2A). HPV did not appear to accelerate syndecan shedding as reported for several bacterial pathogens [32].

To characterize HPV16 released from cells, media from virus-exposed cells were concentrated with Amicon 30 ultra-filters then applied on a Sepharose 4B column. This method is used widely to isolate and characterize differently sized complexes [33]. Size-exclusion chromatography fractions were analyzed by SDS-PAGE and immunoblot. As expected based on the large MW of the virus, HPV16 eluted in void volume fractions of this highly porous gel (fraction 4 contains large complexes or particles with MW>10^7^ Da) [34] (Figure S2). Immunoblot analysis of void volume fractions revealed that the amount of HPV released into the medium increased with time (Figure 2B). There are at least two explanations for release of bound HPV16 from the cell surface in CM. First, the non-covalent association of HPV to HSPG is dynamic and viral particles could dissociate from the cell and associate with soluble high concentrations of competing syndecan-1 in the serum-containing CM. Second, HPV could be released in complex with syndecan-1 or HS *via* the activity of MMP cleaving the anchored ectodomain of the HSPGs or by heparinases liberating HS. The second scenario is consistent with our finding that syndecan-1 and HPV16 are released in Tyrode's buffer, which is devoid of soluble syndecan (Figure 2A and not shown, respectively). If HPV exposure leads to increased MMP activity, gelatin zymography analysis should reveal a higher level of gelatinases in experimental medium in the presence of virus. However, this sensitive and widely used method detects non-active latent MMP forms in addition to active forms [35], and failed to show a change in MMP levels when virus was present (Figure 2C). Therefore, to more specifically test the involvement of MMP activity in HPV release and infection, we investigated the effects of MMP inhibitors on virus release and infection. The large number of MMPs important for epithelial cell HSPG ectodomain shedding and fact that many posses overlapping substrates *in vitro* make genetic knockdowns unfeasible [36]. Since we wished simply to determine if HSPG release was related to HPV16 infectivity, we tested broadly active MMP inhibitors, batimastat (BM) marimastat (MM), and tissue inhibitor of metalloproteinases 3 (TIMP3) that are typically used to assay the functional consequences of inhibiting MMP activity and the release of their substrates [25], [31]. Whereas TIMP3 broadly inhibits ADAM-TS4 and ADAM-TS5 and all MMPS tested to date, BM is specific to MMP -1, -2, -3, -7 and -9; MM blocks function of MMP -1, -2, -7, -9, -14. These widely used hydroxamic acid MMP inhibitors are well known to block the release of syndecans from cells when used at >1 µM concentrations [37]–[39]. Both BM and MM effectively prevented the release of HPV16 from cells (Figure 2D,E) and efficiently reduced HPV16 infection of HaCaT cells (Figure 2F). TIMP3 also prevented HPV16 infection (Figure 2F), but due to lower MMP specificity was not investigated in other assays. A dose-response analysis of BM and MM revealed HPV16 infection inhibition at an IC_50_ of 400 nM (BM) and 1 µM (MM) in the absence of visible toxicity (Figure S3). Thus, the actions of the inhibitors indicate that MMPs are involved the release of virus from cell membranes and that virus release plays an important role in infection.

### HPV particles released in HMW complexes are associated with syndecan-1, HS and growth factors

**Figure 3:**
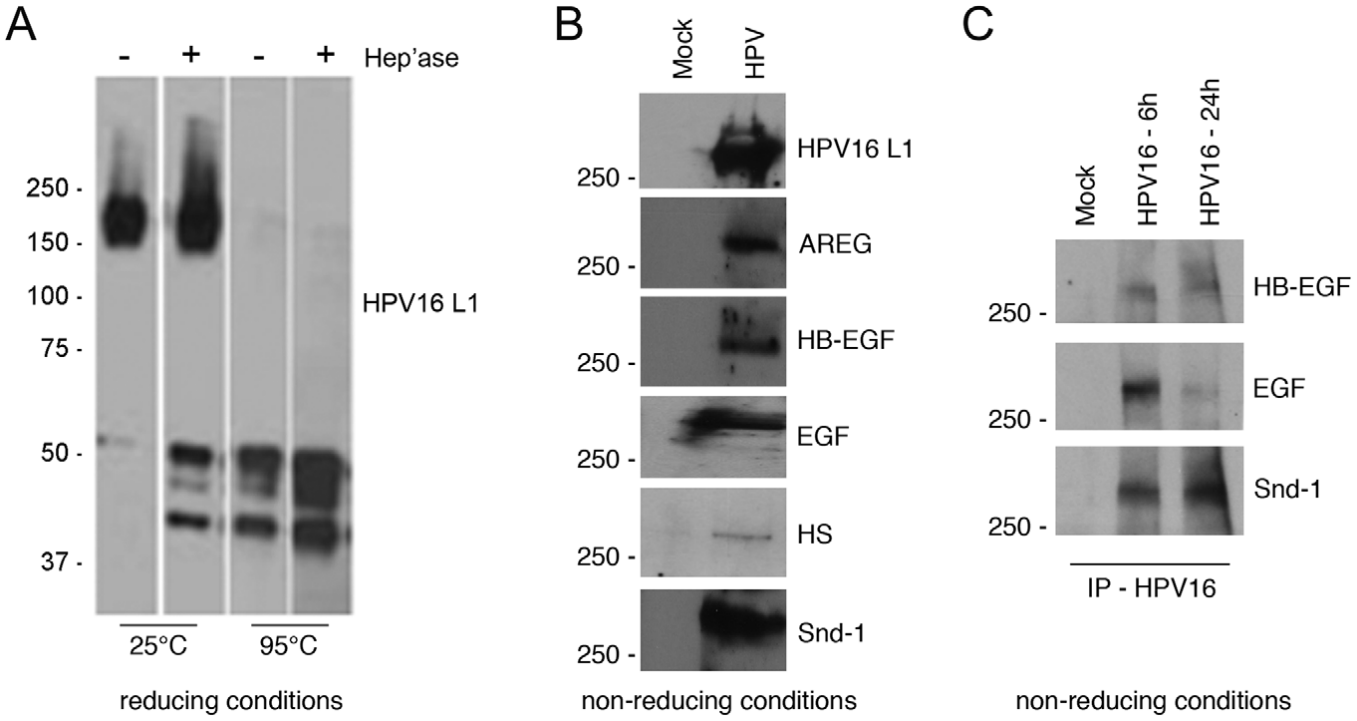
Sepharose 4B gel chromatography of media constituents from HPV-exposed HaCaT cells. Sepharose 4B chromatography was performed on released components in the CM of HaCaT cells exposed to HPV16 PsV for 4 h; see Figure S2. (A) The void volume (HMW) fraction was divided into four parts that were untreated or incubated with 1 U heparinase III for 2 h at 37°C then solubilized in 6× sample buffer and incubated at 25°C or boiled for 7 min before SDS-PAGE and L1-immunoblot analysis. Separate lanes shown are from the same exposure of the same film. (B) Non-reducing SDS-PAGE of void volume Sepharose 4B fractions from released components in the CM of HaCaT cells mock-exposed or HPV16-exposed for 24 h. Immunoblot analysis was done to detect L1, amphiregulin (AREG), HB-EGF, EGF, HS and syndecan-1 (snd-1). (C) Non-reducing SDS-PAGE of released components following IP of HPV16 from CM of HaCaT cells mock- or HPV16-exposed. HPV16 exposed cells were allowed to bind virus at 4°C for 1 h, washed to remove unbound particles, then shifted to 37°C for 6 h or 24 h. CM were subjected to IP for HPV16 using affinity purified polyclonal anti-HPV16 VLP antibody covalently attached to magnetic beads. Immunoblot analysis was performed to detect HB-EGF, EGF, and syndecan-1.

Syndecan HSPGs participate in assembling signaling complexes by accumulating biological mediators including GFs and presenting these factors to their high affinity receptors [40]. Therefore, we predicted that released HPV particles would be in complex with HS (or HSPG) of varied sizes along with assorted GFs. Solubilization of the Sepharose 4B void volume fraction (MW>10^7^ Da) in SDS-mercaptoethanol sample buffer and boiling caused dissociation of virus resulting in a ∼55 kDa band of HPV16 L1 protein (Figure 2B). We found temperature to be crucial for viral complex dissociation; without heating, HPV16 L1 in SDS-reducing buffer was detected only in a form >150 kDa (Figure 3A). These results indicate the cell surface-released HPV is part of a detergent-resistant and temperature-sensitive HMW complex. To determine the role of HS in this complex, the Sepharose 4B void volume fraction was exposed to heparanase III. Treatment with heparanase III induced partial dissociation of HMW complexes and a considerable amount of soluble HPV16 L1 was detected at ∼55 kDa, indicating that HS is involved in formation of HMW virus-containing complexes. Under non-reducing conditions in HMW fractions, HPV16 L1 migrated well above 250-kDa (Figure 3B) demonstrating the reducing conditions caused dissociation of some complexes. This is in contrast to the fact that L1 proteins from purified mature HPV PsV appear as 125 kDa dimers and 195 and 215 kDa trimers under non-reducing SDS-PAGE conditions, but never migrate above 215 kDa [41].

Next we used the HMW void volume Sepharose 4B fraction for analysis of GFs and HS. Individually these molecules are low molecular weight and mainly elute from the column in later fractions (>9, Figure S2B). Fractionated media from mock exposed HaCaT cells was a control. Immunoblot revealed the presence of amphiregulin (AREG), heparin binding epidermal growth factor (HB-EGF), EGF, HS, and syndecan-1 but only in HMW fractions of media from cells exposed to HPV16 (Figure 3B). Non-reducing SDS-PAGE of this void volume Sepharose 4B fraction showed all of these molecules were present, each appearing to be ≥250 kDa in size. To more specifically assess the direct association of these components, we performed an IP for HPV16 particles released into CM following virus binding to cells at 4°C and shift to 37°C for 6 or 24 h. Immunoblot for HB-EGF, EGF and syndecan-1 demonstrated these factors were in a complex with HPV16 released from cells (Figure 3C). These findings indicate HPV particles released in HMW complexes from cells are “decorated” with syndecan-1 ectodomains, HS, and assorted GFs. Although post-attachment release of incoming virus has been reported for some retroviruses [42]–[45], to our knowledge this is the first demonstration of an attached incoming non-enveloped virus being liberated from the cell surface into the experimental medium. Further, this is the first report of a mechanism, distinct from dissociation, by which bound virions are released from cells.

### Released HPV16 complexes are infectious and HSPG play a crucial role in infection

**Figure 4:**
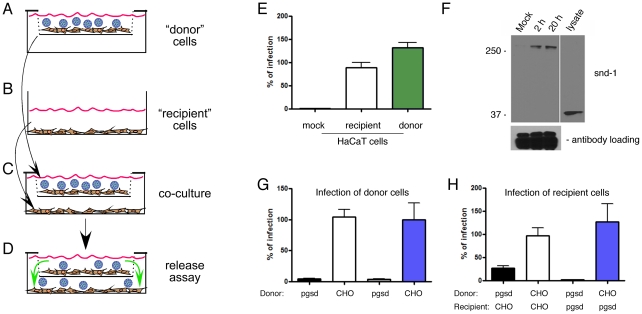
Released HMW complexes including HSPG and HPV16 are required for infection. (A–D) Schematic of the “donor” cell/“recipient” cell co-culture system indicating how cells were exposed to PsVs. PsVs were allowed to bind donor cells without internalization (A). Donor cells on coverslips were washed thoroughly to remove unbound PsVs and transferred to mesh inserts above the recipient cells (B) to co-culture with gentle rocking for 24 h and allow released HPV complexes from donors to access the recipient cells (C–D). All experiments employed CM. (E) Relative HPV16 infection levels of HaCaT donor cells and recipient HaCaT cells compared to mock infected cells as verification of the co-culture virus release model. (F) Non-reducing SDS-PAGE and immunoblot of syndecan-1 (polyclonal rabbit antisera) following IP of HPV16 (mouse monoclonal anti-L1). IP was performed by immobilizing anti-L1 in the lower chamber in place of cells (see panel B) to capture HPV16 released from mock exposed cells (M) or HPV16-exposed HaCaT donor cells at 2 or 20 h post virus exposure. Lower panel IgG detection is included as a loading control. (G) Relative infection levels in CHO-K1 and pgsd-677 cells used as donor cells bound to HPV16 PsV and co-cultured above the recipient cells. (H) Relative infection levels in CHO-K1 and pgsd-677 cells used as recipient cells co-cultured below the PsV-bound donor cells corresponding to the data in panel G. Infectivity data (E,G,H) were normalized to the mean value of the infected control set to 100% and represent the mean ± SEM of 4 replicate infections.

To ascertain if released virus complexes were infectious, we designed a co-culture transwell system wherein unexposed (“recipient”) cells were cultured in chambers below an insert holding “donor” cells that separately had been exposed to HPV16 (Figure 4A–D). As a proof-of-principle, HaCaT cells were tested as both donor cells and recipient cells (Figure 4E). HaCaT donor cells were allowed to bind HPV16, washed to remove unbound virus and placed atop recipient HaCaT cells where they were incubated with gentle rocking for 24 h. Comparable infection levels were detected between directly PsV-exposed HaCaT donor cells and the recipient HaCaT cells grown in the lower chamber demonstrating the infectivity of the released HPV16 material (Figure 4E).

To verify that HPV16 released from donor cells was in a complex with syndecan-1, we used bead-attached anti-HPV16 antibody instead of recipient cells in the lower chamber. Following capture of the viral particles, non-reducing SDS-PAGE and immunoblot for syndecan-1 confirmed the co-IP of syndecan-1 with released HPV16 (Figure 4F). Similar to when the material released into cell media was subjected to chromatography (Figure 3), the syndecan-1 plus L1 complex released from donor cells appeared as a HMW form ≥250 kDa. Conversely, only the 35-kDa monomeric form of syndecan-1 was detected *via* this rabbit antiserum in the cell lysate from cells not exposed to HPV (Figure 4F).

To determine the importance of HS in the infectious process following PsV release, we tested wild-type Chinese hamster ovary (CHO-K1) cells and mutant CHO cells defective in HS biosynthesis (pgsd-677) [46]. Consistent with previous reports [18], [47], we found the HSPG-defective cells could be infected by HPV16 PsV, but at levels reduced to only ≈5–8% of the wild-type CHO cells (Figure 4G). Using our co-culture system, PsV-exposed CHO-K1 or pgsd-677 donor cells were placed atop of CHO-K1 or pgsd-677 cells grown as recipient cultures. Infections were assayed in paired donor and recipient cells from these co-cultures (Figure 4G and H, respectively). Donor CHO-K1 cells exposed to HPV16 PsV could fully confer infection to recipient CHO-K1 cells (Figure 4H, white bar). Importantly, recipient pgsd-677 cells were also fully able to support infection, but only when CHO-K1 cells were used as PsV donors (Figure 4H, blue bar). These results demonstrate for the first time that HSPG attachment receptors are not required for recipient cell infection when HPV particles are released in complex with HSPG from donor cells that are able to express HSPG. These data show an essential infectious role for the released HMW complexes containing HPV16 decorated with HS on cells that lack HSPG. That donor pgsd-677 cells could confer limited infection to CHO-K1 cells (Figure 4H, black bar) may reflect low level dissociation or release of virus to the fully receptive HSPG-wild type CHO-K1 cells.

### HPV PsVs interact with growth factor receptors

**Figure 5:**
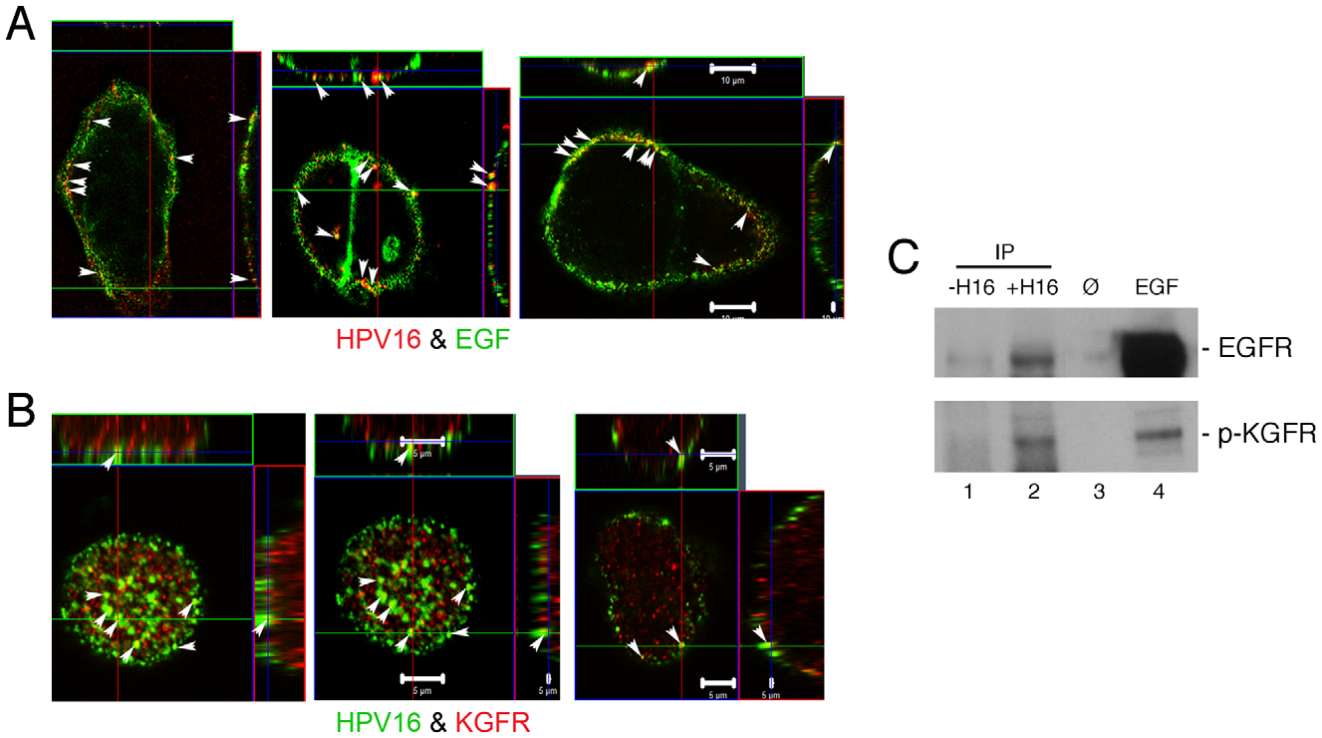
HPVs interact with growth factors and growth factor receptors on human keratinocytes. Immunofluorescent confocal co-localization (arrowheads show examples of signal overlap) showing top view and side views of non-permeabilized HaCaT cells. (A) Co-localization of HPV16 (red) with EGF (green). Bars measure 10 µm. (B) Co-localization of HPV16 (green) with KGFR (red). Bars = 5 µm. (C) SDS-PAGE and immunoblot for EGFR or p-KGFR after IP of HPV16 from HaCaT cells exposed to PsV. Lane 1, HaCaT cell lysate (without HPV exposure) incubated with anti HPV antibody attached to magnetic beads (negative control); lane 2, IP of HPV16 (mouse monoclonal anti-L1) from virus-exposed HaCaT cells; lane 3, blank; lane 4, HaCaT cell lysate following EGF exposure as positive control.

We hypothesized that if specific GFs were present in association with HS-decorated virus, the very high affinity of GFs for their specific receptors (*K*_D_≈10–100 pM) might permit the GF to determine the fate of the virus-cell interaction prior to HPV entry. If supported, we should detect interaction of virus with GF receptors (GFR). Co-localization of HPV16 with GFs and GFRs was assayed by confocal microscopy and physical associations were tested by co-IP. HaCaT cells exposed to HPV16 were either incubated with fluor-labeled EGF or immunostained for KGFR. Figure 5A,B shows the partial co-localization of HPV16 with EGF and KGFR on the cell plasma membrane. IP of HPV16 PsVs provided additional evidence of interactions with EGFR and KGFR following PsV binding to HaCaT cells. Immunoblot demonstrated the co-IP of EGFR and phospho-KGFR from HaCaT cells following the IP of HPV16 PsVs (Figure 5C). These data confirm the interaction of HPV16 with EGFR and KGFR on the plasma membrane of human keratinocytes. The chromatography and IP data together support the idea that HPV particles become decorated with HS and bioactive molecules like GFs to interact with GFRs.

### HPV exposure induces rapid GFR phosphorylation and activation of downstream effectors

**Figure 6:**
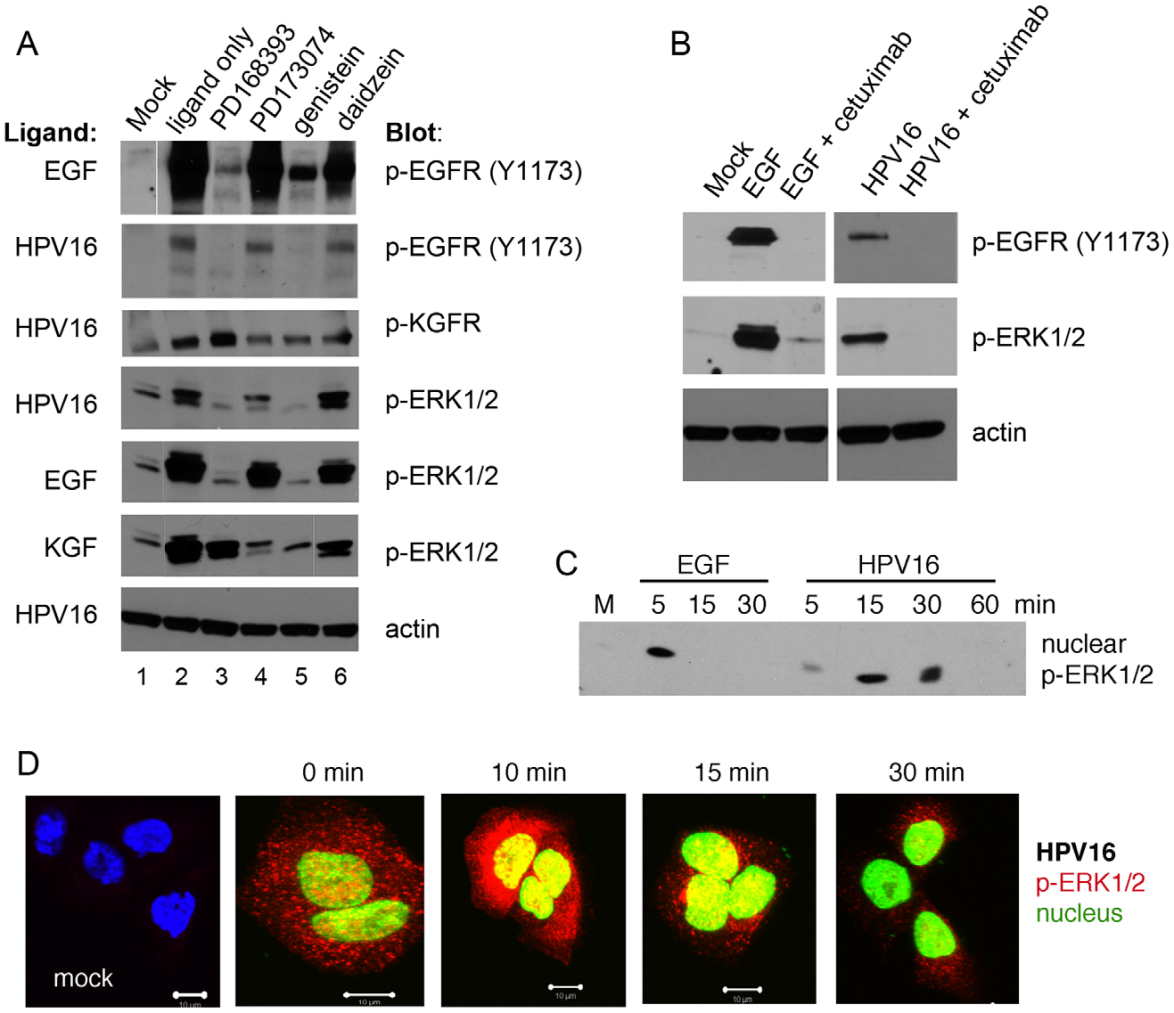
HPV16 activates EGFR and KGFR signaling pathways. (A–B) Immunoblot for p-EGFR, p-KGFR and downstream effector p-ERK1/2 following 10-min ligand exposure (lane 2; listed at the left of each blot: EGF, KGF, HPV16 PsV) in HaCaT cells serum-starved for 4 h. Mock exposed cells were negative controls (lane 1); ligand controls included 10 ng/ml EGF and 10 ng/ml KGF; HPV16 PsV dose was 20 vge/cell. Cells were pretreated with the indicated inhibitors in Tyrode's buffer (100 nM PD168393, 1 µM PD173074, 100 µM genistein, 100 µM daidzein, 600 nM cetuximab) and exposed to the ligands in the presence of inhibitors Tyrode's buffer. Actin is detected as a loading control. (C–D) Immunoblot of nuclear cell fractions and confocal microscopy localization of p-ERK1/2 following EGF and HPV16 exposure in serum-starved HaCaT cells at indicated times post-exposure. (C) Immunoblot for p-ERK1/2 in nuclear fractions from exposed cells. (D) Immunofluorescent confocal microscopy for localization of p-ERK1/2 (red) in PsV exposed cells. DAPI was used as a nuclear marker and was pseudocolored green to facilitate efficient co-localization of p-ERK1/2 in the nucleus. Parameters of lasers intensities were kept constant during the imaging.

The engagement of GFRs by their ligands induces rapid auto-phosphorylation and downstream signaling. To investigate the involvement of EGFR and KGFR activation and signaling in HPV infections, we analyzed phosphorylation levels of the GFR and mitogen-activated protein kinases (MAPKs) ERK1/2, key enzymes of their pathways [29], [48]. HaCaT cells starved in SFM for 4 h were incubated with low doses of HPV PsV (10–20 vge/cell) to avoid non-specific events; phosphorylation of target proteins was determined by immunoblot analysis. Consistent with receptor-ligand kinetics, GFRs were rapidly activated within 10 min of treatment with ligands (GFs or HPV16) inducing concomitant phosphorylation of the downstream effector ERK1/2 (Figure 6A). Phospho (p)-EGFR (Y1173) levels induced by HPV16 were considerably lower compared to the effect induced by EGF. The Y1173 site of EGFR is involved in MAPK signaling, and importantly, the phosphorylation levels of p-ERK1/2 induced by HPV16 were comparable to the effect of EGF (Figure 6A).

Treatment with a potent inhibitor of EGFR (PD168393), a pan-FGFR inhibitor (PD173074), or the general receptor tyrosine kinase (RTK) inhibitor, genistein, before exposure to GFs or HPV16 diminished the rapid phosphorylation of the target GFRs and downstream p-ERK1/2. KGFR activation of ERK1/2 can involve EGFR cross talk and activation [49], [50], which may explain why EGFR inhibitor PD168393 fully blocks ERK1/2 activation by HPV16 when it also appears KGFR signaling is initiated by the virus. In contrast, daidzein, a genistein analog that lacks RTK blocking activity, did not inhibit HPV16-induced signals (Figure 6A). To specifically query ligand-dependent EGFR activation by HPV16, we investigated the effects of cetuximab, an EGFR-specific monoclonal antibody that binds to the EGFR extracellular domain with a higher affinity than ligands EGF or TGF-alpha. Cetuximab inhibits EGFR phosphorylation and activation and leads to receptor internalization and degradation [51]. We found that cetuximab fully abrogated EGF- and HPV16-induced phosphorylation of EGFR and p-ERK1/2 in this assay (Figure 6B).

GFs strongly activate ERK1/2 proteins [52] and upon stimulation, a significant population of these kinases moves from the cytoplasm into the nucleus [53]. P-ERK1/2-specific immunoblotting of nuclear protein fractions and confocal microscopy each revealed nuclear movement of p-ERK1/2 upon virus-induced activation (Figure 6C,D). The timing of the p-ERK1/2 nuclear migration induced by HPV16 exposure reached maximum ≈10 min post exposure and indicates signaling pathways are activated as early as 5 min post virus-host interaction. These results agree with the report showing that even low-level EGFR activation can fully induced ERK1/2 signals in human keratinocytes [54].

### GFR inhibitors hinder HPV infection

**Figure 7:**
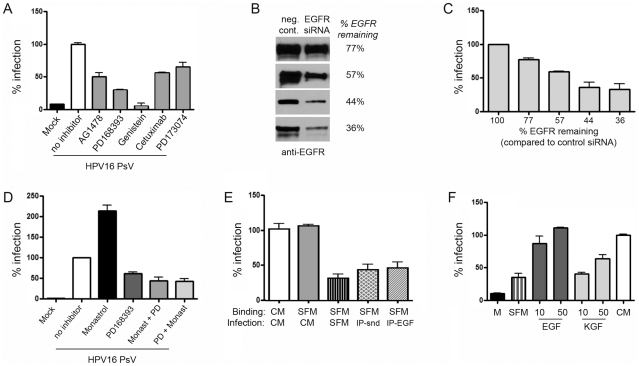
GFR activation, EGFR expression levels, and serum components including GFs are important for HPV16 infections. (A) Relative HPV16 infection of HaCaT cells in the presence of specific GFR and protein tyrosine kinase inhibitors. Subconfluent HaCaT cells were pre-treated 45 min with 1 µM AG1478, 100 nM PD168393, 100 µM genistein, 100 µM daidzein, 1 µM PD173074, or 100–600 nM cetuximab. Cells were exposed to HPV16 PsV at 100 vge/cell for 1 h at 4°C, then washed extensively and shifted to 37°C in the presence of the indicated inhibitor in CM for 24 h at which time they were analyzed for luciferase expression. Data are represented as mean ± SEM of 3 experiments. (B–C) EGFR knockdown in EGFR-siRNA transfected HaCaT cells was determined by immunoblot and compared by densitometry to EGFR levels in cells transfected with a negative control siRNA at 48 hours post transfection. Four separate transfections were analyzed (B) and HPV16 PsV infection levels were measured at 24 h post infection (48 h post transfection) (C). Error bars represent the average of triplicate luciferase readings from the four transfections. (D) HPV16 PsV infection levels (24 h post infection) in the presence of inhibitors following pre-treatment for 1 hr with 100 µM monastrol, pre-treatment with monastrol for 1 h plus 500 nM PD168393 for duration (monast.+PD), or pre-treatment with 500 nM PD168393 for 1 hr plus 100 µM monastrol for duration (PD+monast.). (E) Relative HPV16 infection is dependent upon medium constituents post primary HPV16 binding. HaCaT cells starved in SFM (4 h) were exposed to HPV16 in CM (positive control) or SFM. After washing away unbound virus, cells were incubated for 24 h in CM, SFM, syndecan-1-depleted CM (IP-snd), or EGF-depleted CM (IP-EGF). Infections were quantified by luciferase assay at 24 h post shift to 37°C. Data are represented as mean ± SEM of 3 experiments. (F) Relative HPV16 infection in SFM is enhanced by GFs. HaCaT cells starved in SFM were exposed to HPV16 in SFM for 1 h at 4°C. After washing away unbound virus, cells were incubated for 24 h in SFM, SFM containing GFs (concentrations indicated: ng/ml), or in CM. Infections were quantified by luciferase assay; bars represented the mean ± SEM of ≥3 individual experiments.

To evaluate the importance of GFRs and tyrosine kinase activation in HPV infection, HaCaT cells were incubated with HPV PsV following pretreatment with and in the presence of a reversible (AG1478) or an irreversible (PD168393) EGFR-specific inhibitor, genistein, cetuximab, and an FGFR inhibitor (PD173074) in CM. Both EGFR specific biochemical inhibitors substantially blocked infection by HPV16 (≥50%), while genistein almost completely inhibited infection (Figure 7A). Treatment of HaCaT cells with an EGFR blocking antibody (cetuximab) or FGFR/KGFR inhibitor (PD173074) reduced infectivity by 50 and 35%, respectively. Similar GFR signaling activation and response to inhibitors was observed with HPV31 PsV and with particles carrying the viral genome (not shown; [55]), ruling out a luciferase-specific inhibition. The complete inhibition of HPV infection by preventing RTK signaling with genistein demonstrates the requirement for this class of receptors in HPV infection. Specific inhibitors of EGFR (cetuximab, AG1478, PD168393) or of KGFR (PD173074), while completely abrogating signaling from their respective RTK under brief starvation conditions described in Figure 6, only partially reduced HPV infection under conditions in CM (Figure 7A). These data show that no single RTK is essential for HPV16 infection of HaCaT keratinocytes; rather, EGFR, KGFR, and potentially other RTK are important mediators of HPV infection. A genetic approach using siRNA to inhibit EGFR expression gave complementary results. Typical transfection efficiency of HaCaT cells was ≈70% as monitored by fluorescein-labeled control siRNA. EGFR knockdown was assessed by immunoblot in four separate transfections at 48 h post transfection and ranged from remaining EGFR expression of 77% to 36% compared to cells transfected with a nonspecific control siRNA (Figure 7B). HPV16 PsV infections were performed 24 h post transfection in matching replicates. Infection levels measured 24 h later were reduced in a dose-dependent manner that closely paralleled the level of EGFR knockdown (Figure 7C).

Because progression into early M-phase is needed for HPV infection [56], it was important to assess whether the inhibitors prevented infection *via* cell cycle blockade. Therefore, we assayed the fraction of cells in each phase of the cell cycle during the inhibitor treatments under which infections were determined above. Although every condition affected the cell cycle distribution, in no case did an inhibitor arrest the cells in any one cell cycle phase. Further, we found no correlation between infection inhibition and cell cycle distribution under the assay conditions employed (Figure S4). For example, the distribution of cells in the G1, S, or G2/M phases of the cell cycle were relatively similar whether cells were grown in CM and infected with HPV16 with no treatment or treated with batimastat, marimastat, PD173074, PD168393, or cetuximab. However infection levels ranged from 0% decrease with no inhibitor to nearly 90% reduction with marimastat (Figure 2E). Specifically, the moderate changes observed in the number of cells in G2/M phase were not sufficient to account for the levels of infection inhibition demonstrated for each inhibitor tested. The most striking result was found when using monastrol, which increases the number of cells in late M-phase and promotes infection [56] (Figure 7D). When PD168393 treatment was added with monastrol, a similar cell cycle profile was seen, yet infection was dramatically inhibited by ≈70% (Figures S4 and 7D, respectively). These data indicate that cell cycle effects cannot account for the inhibition of early infection events by these various compounds.

### Serum enhances HaCaT cell infection with HPV

The cell binding and infectivity of some viruses are affected by medium composition [57], [58]. We also found HPV infection of HaCaT cells to be dependent upon the nature of the experimental media. Equal doses of HPV16 were allowed to attach to serum-starved cells in SFM at 4°C and, after washing away unbound virus, cells were incubated at 37°C overnight in SFM or CM. As a positive control HaCaT cells were used where virus binding and infection were both performed in the presence of CM. As shown in Figure 7E, there was no difference in infection levels between the positive control and cells where virus was bound to cells in the presence of SFM and thereafter incubated with CM. This demonstrates that virus binding to initial attachment factors is unaffected by the nature of the media. The addition of serum (containing various GFs and HSPGs) to media significantly increased virus infection, indicating an important role for these molecules in virus uptake and infection. As we found that CM contains considerable amounts of syndecan-1, likely in complex with GF [40], we predicted depletion of syndecan-1 from the CM would remove a substantial level of components needed for infection. As expected, when the CM was stripped of syndecan-1 by IP, infection levels were reduced to levels close to those in SFM (Figure 7E). Similarly, depletion of EGF from the serum also robustly reduced infection levels (Figure 7E).

Based on our finding that bound HPV particles become decorated with HS and are released from cells plus the fact that main constituents of serum include albumin and GF, we performed the reciprocal experiment and tested whether GFs facilitate infection. If GFs are responsible for bridging the soluble HMW HPV-HSPG complexes to secondary receptors, then reconstituting GF in SFM should restore infectivity. Although the addition of albumin did not enhance infectivity in SFM (not shown), the addition of EGF and KGF in SFM dramatically restored infection in dose dependent manners. EGF was able to fully restore infection levels but KGF at the same concentrations was only able to partially restore infection levels to those seen in CM (Figure 7F). Thus, we show syndecan-1 plus either EGF or KGF are required for HPV16 infection of human keratinocytes. Although infection in SFM increased the number of cells in G1 phase, the depletion conditions did not alter the cell cycle profiles significantly from that in CM or in SFM plus EGF (Figure S4), suggesting cell cycle changes alone could not account for infection inhibition.

## Discussion

Intracellular pathogens like viruses hijack many normal cellular processes in order to gain entry into a host cell [5]. Some viruses have multiple structural proteins that are required to initiate cellular uptake, whereas other viruses use one or two viral capsid proteins for interaction. Certain viruses bind directly to uptake receptors, whereas others first bind to cellular attachment factors that are generally thought to lack specificity before particles are laterally transferred to internalization receptors. In several cases, early binding events may trigger capsid conformational changes that permit virion movement to and/or interaction with an entry receptor, dictate signaling to initiate endocytosis, and/or activate membrane fusion activities for some enveloped viruses. Although a variety of cellular interacting factors have been identified for the HPV infection process, many specifics of the early stages of HPV-cell interaction have been enigmatic. HPV particles engage HSPG attachment moieties and are thought to dissociate from HSPGs or to move laterally to interact with secondary receptors that promote endocytosis. Yet, the mechanism facilitating virus movement from primary attachment to the internalization receptor(s), or whether the process is spontaneous or highly controlled, has not been defined.

Syndecan-1, the predominant HSPG in keratinocytes, is a demonstrated primary HPV-cellular interacting partner [18], [19]. The HPV-HS interaction was first thought to be nonspecific, but recent reports show that HS modifications by sulfate groups are essential for HPV types 11, 16 and 33 capsid interactions with cells [14], [59]. HPV L1 proteins mediate the capsid binding to HSPG; L1-only VLP are capable of normal cellular internalization [16] and the L2 protein does not contribute to the initial interaction [60]. It has been proposed that L1-HSPG binding induces conformational changes in the viral capsid that cause the normally hidden N-terminal region of L2 to become accessible to furin cleavage [6]. This action on L2 is suggested to trigger reduced affinity of capsids for HS [61]. However, the ability of L2 cleavage to induce an L1 conformational change of the magnitude that would cause dissociation from this strong interaction with HS is difficult to envision and prompted us to investigate the means of HPV movement from HSPG to secondary receptors.

Here we report evidence for a novel mechanism by which a virus commandeers a normal cellular process, in this case to transfer from general attachment factors to receptors responsible for infection (Figure 1B). Our results show that HPV16 is not dissociated from syndecan-1 HSPG, but is released from the cell surface in complex with a shed form of HSPG that also carries GFs and/or other bioactive compounds (Figure 1B*iii*). Although the entirety of this transfer mechanism is a unique observation with regard to viruses, various aspects of this process have been reported for other intracellular pathogens. For example, infecting retroviruses are shed from a cell-attached state, which, based on their association with HSPGs, could be induced by the same mechanism of cellular liberation we show here [42]–[45]. *Chlamydia trachomatis* interacts with HSPGs for primary attachment to cells and also with fibroblast growth factor 2 (FGF2) to promote bacterial entry [62].

Extracellular domain shedding of proteins is involved in the control of diverse cellular functions such as development, growth, differentiation, and wound healing as well as various pathologies like cancer [21], [23], [25], [28], [63]. For example, it has recently been recognized that ectodomain shedding functions to control the availability of EGFR ligands, TGF-beta receptor, TNF-alpha, and cell adhesion molecules (L-selectin, E-cadherin). Members of the EGF family are synthesized as a type-1 transmembrane protein that can be enzymatically cleaved to release soluble 14–20 kDa GFs (Figure 1A*i*). Most of these soluble GFs are then concentrated on the HS chain of proteoglycans (for example syndecans) and shedding of these HSPGs appear to play modulatory roles, such as by presenting the GFs to their high affinity cognate receptors to activate signaling and receptor-ligand endocytosis [23], [24]. Ectodomain shedding of membrane-bound proteins is mediated by proteases known as sheddases. Among these enzymes, MMPs are the predominant syndecan sheddases, but several other factors like heparinases act cooperatively to regulate this process [21]. Here we show that release of cell–bound HPV is regulated by MMPs and inhibition of sheddase activity significantly decreases virus release and infection. MMPs have been implicated in neoplastic situations for some time, with several reports suggesting that HPV early proteins play a role in the regulation of MMP expression and activity [64]. The relationship between MMPs and HPV proteins appears to be complex and further work will be necessary to fully appreciate their interactions.

Following what we term as the HPV decoration process, whereby viral particles associate with HSPG-bound GFs and are liberated from cells *via* normal sheddase activity, the decorated HPV particles act as soluble effectors of infection (Figure 1B*iii*). The specificity of the GF or other bioactive molecule is used to bridge and interact with the cognate cellular receptor (i.e., a RTK/GFR) and induce signals needed for initiation of infection (Figure 1B*iv*). As soluble HPV particle could be decorated with various active molecules, there is no single RTK responsible for internalization. Herein, we concentrated our attention on EGFR and KGFR due to their important regulatory roles in keratinocytes. Our results clearly define activation of GFRs as a necessary step in infection. Both of these receptors are rapidly activated by interaction of HPV16 with HaCaT cells, and varied specific inhibitors of the receptors block their phosphorylation. Moreover, rapid phosphorylation of key downstream effectors of these pathways (ERK1/2) was observed. This is in agreement with recently published data showing fast activation of PI3K and FAK kinase upon HPV16 PsVs binding to HaCaT cells [65], [66]. Although we did not directly measure PI3K and FAK activation, they are upstream of ERK1/2 in the signaling pathway and play important roles in the regulation of these MAPKs. Specific inhibitors of EGFR and FGFR only partially inhibit infection, whereas genistein (a general tyrosine kinase inhibitor) completely blocked infection of HaCaT cells with HPV16. These data support our conclusion that there is no sole pathway essential for HPV16 infection; the virus could infect cells using multiple pathways and receptors. Regulation of GFR activity during persistent HPV infections is well known, and this is attributed to actions of virus early proteins in infected cells [67], [68]. Here we demonstrate that even in the earliest stages of HPV-host cell interaction, and prior to viral gene expression, oncogenic HPVs usurp mitogenic GFR signal pathways that cause nuclear localization of ERK1/2. Importantly, these signals cause activation of AP1 transcription factors, c-fos and c-jun, which are important for HPV early transcription and are thought to dictate the strict epithelial tropism demonstrated by HPVs [69]–[71]. In this way, HPV interaction at the cell surface, like that of many other viruses, primes the host cell for viral gene expression and the establishment of infection.

The goal of this work was to determine how HPV moves from HSPGs to initiate virus entry and we focused our analyses at the plasma membrane. Although we have not unequivocally shown GFRs to mediate virus endocytosis, several lines of evidence support the idea that the GFRs can facilitate HPV entry. First, we show HPV16 PsVs reside in a complex containing HSPGs and GFs, and can activate GFR signaling in human keratinocytes soon after PsV exposure. HPV particles associate with GFRs at the keratinocyte plasma membrane and the removal of syndecan-1 HSPG or EGF inhibits infection. These observations strongly indicate the virus interacts with GFRs to physically induce signals and that this is not an indirect effect of virus exposure. Secondly, interaction of bioactive HS-GF with their specific receptors typically leads to receptor-mediated endocytosis following the signals activated [26]. Both GFR signaling and HPV infection are reduced using a variety of RTK inhibitors and a genetic knockdown approach. Lastly, many other intracellular pathogens can use GFRs as internalization receptors, some *via* physical bridging of GFs to their cognate receptors. EGFR can be used as an entry receptor for vaccinia, cytomegalovirus, herpes simplex, influenza virus, and hepatitis C virus [72]–[77]. Yet, in many cases – just as we show for HPV16 – EGFR is not the sole entry receptor for these pathogens. Furthermore, *Chlamydia trachomatis* uses FGF2 to engage the FGFR for uptake and a herpes simplex virus engineered with an EGF ligand can bridge to cognate GFRs to activate entry [62], [78].

Our new model for the HPV infection process shown in Figure 1B incorporates findings from a number of prior studies. Importantly, our model facilitates the explanation of some discrepancies in the literature regarding HPV-cell interactions and entry. These include the nature of secondary receptor interactions, disparate virus internalization rates and pathways, and differences between infectivity of the viral particles obtained from organotypic (raft) tissues and virus particles obtained from the 293T expression system. Lastly, our findings fit well with the observed importance of wounding in HPV infections, and may have broader implications for pathogen-cell receptor interaction *via* HSPGs and GFs.

### Nature of the secondary receptor interactions, virus internalization rate and pathways

Integrins, laminin 332 and syndecans have all been shown to interact with HPVs [19], [65], [79], [80]. Each of these interactions may be primarily due to the association of HPV particles with HSPGs, which are direct modifiers of syndecan-1 and interaction partners with laminin 332 and alpha-6 integrin (as shown in Figure 1). We demonstrated HPVs associate with HS molecules bearing various GFs and interact with EGFR and KGFR. Together, our findings indicate that binding of HPV to a secondary receptor depends on the nature of active compounds decorating HPV.

Reported entry half times for HPVs range from 4 h to 24 h [3], [14], [15], [18], [81], [82]. Although we reported a 14 h internalization half-time for HPV31 in HaCaT cells [82], we also detected HPV31 early transcripts by RT-PCR as early as 4 h post infection [83]. These observations suggest that some HPV particles are able to enter *via* an infectious route much more quickly than others. The findings in this current study and the normal biology of HS-GF complexes lead us to reason that the protracted and variable HPV entry timing is due to the multiple locations and ways that virions can become decorated with HS-GF complexes (Figure 1B). Particles decorated with HS-GF during isolation or potentially associating with these soluble materials in serum (Figure 1B*v*) may be readily able to directly engage the entry receptor, effectively bypassing the more time consuming steps of HSPG-GF interaction and subsequent enzymatic release of HMW complexes. Our data and reports from other labs showing RTK/GFR signaling can occur minutes after virus exposure also support this idea [65], [66], [84].

The preferential association of HPV with the ECM and basement membrane appears to be due to interactions with laminin 332 (formerly named laminin 5; Figure 1B*ii*) [17], [80], [85]. This is likely because laminin 332 is a depot for HS-GF complexes to which HPV can attach [86], and these active complexes can be liberated by heparinases and sheddases [87]. Our co-culture assay does not differentiate between virus released from the cell surface or the ECM. We previously reported the disappearance of ECM-bound HPV over time [55] suggesting that the release of both ECM- and plasma membrane-bound HPV-HS-GF complexes could contribute to the infectious process. Thus, longer internalization kinetics would be expected if some HPV capsids associate with HS-GF by binding HSPG on the plasma membrane, or by associating with the HS-GF complexes that are normally sequestered on the ECM or the basement membrane. MMP- or heparinase-mediated release of these HWM HPV-HS-GF complexes would be required for subsequent engagement of the secondary receptor (Figure 1B*iv*).

We propose the spectrum and diversity of the active compounds (e.g., GFs) with which HPV-HS could interact clarifies why a single secondary receptor responsible for virus internalization has not been identified. Various active compound-virus complexes bind to distinct receptors and consequently are internalized *via* different endocytic pathways, which explains the internalization of HPVs dependent on clathrin [15] or relying on caveolin [82], [88], as well as pathways independent of both clathrin and caveolin [89]. EGFR and KGFR internalization are typically clathrin-dependent. However, EGFR entry can also involve slower clathrin-independent modes and EGFR associates with caveolae and lipid microdomains, especially when coupled with alpha-6 beta-4 integrin [29], [90]. Blocking ligand binding or the kinase activity of these receptors with specific inhibitors clearly shows significant roles for these GFRs in HPV infection. CHO cells lack EGFR ErbB1, but are readily infected with HPVs, further demonstrating the ability of HPV to utilize multiple routes of infection. Similarly, vaccinia virus infection of HeLa cells is EGFR dependent, yet the virus also infects CHO cells using an undefined alternate mechanism [77].

### Differences in HSPG dependence between tissue-derived and 293T system-derived virus preparations

Previously, we showed that organotypic (raft) epithelial tissue-derived HPV31 virions infect HaCaT cells in an HSPG-independent manner [91], whereas HPV31 PsVs from the 293T system are HSPG-dependent in the same cells (our unpublished data and [85]). We speculate that the differences are due to a high level of decoration occurring during virion isolation from the raft tissues, which then allows raft-derived virions to bypass the need for HSPG association on newly exposed naïve cells. This is based on our finding that viral particles extracted from raft tissues are substantially less pure relative to HPV particles obtained from the 293T expression system, likely due to the lower yields of virus particles per cell in the raft system compared to the 293T model [92]. It is probable that low-level HPV capsid decoration occurring during assembly and purification from 293T cells contribute to the basal levels of infection observed in the absence of HSPG or serum components (Figures 4G, 7EF, refs. [18], [47]). Differences in HPV particle decoration due to isolation techniques could result in quantitatively disparate phenotypes depending upon the assays.

The possibility that other structural modifications with functional consequences occur differentially during virion morphogenesis in the raft tissue culture system compared to particle assembly in the 293T system cannot be discounted. Nevertheless, many observations strongly support the biological relevance of differentiation-independent (e.g., 293T cell-derived) HPV particles for functional studies. Self-assembling VLP and PsV capsids containing L1 and L2 are structurally indistinguishable from wart-derived HPV virions [93], [94]. Of specific importance, L1-only HPV VLPs mimic wart-derived virions functionally such that *in vivo* they elicit neutralizing antibodies that confer long-term protection from infection in animal models and in clinical trials [95], [96]. Indeed, these L1-only VLPs are the basis for the successful HPV vaccines in use throughout the world today. Also of particular biological significance, a careful comparison of xenograft tissue-derived cotton tailed rabbit PV (CRPV) virions to 293T-produced CRPV virions established that the virion stocks were essentially indistinguishable as assayed by susceptibility to antibody-mediated neutralization, papilloma induction, and gene expression within lesions in rabbits [97]. HPV PsVs expressed from capsid genes of carcinogenic HPV types like HPV16 have a number of advantages over tissue-derived virions, especially given that virions for carcinogenic HPV types have never been purified in valuable levels from human lesions. High-titer, high-purity PsVs have utility in a wider variety of assays and in more rigorously controlled experiments than the more crude virions obtained from the organotypic tissue culture system [92], [98], [99]. Thus, sound evidence suggests that 293T-derived PsVs provide a functional and practical substitute for working with high-titer carcinogenic HPV virions in many situations [17], [93], [100].

### Implications for *in vivo* infections in a wounded environment

Epithelial wounding, an important mediator of HPV infections *in vivo* [30], leads to the influx and activation of many cell factors shown to interact with HPVs, including those we have identified in this work. GFs, cytokines and chemokines are key mediators of wound repair. EGF and KGF are released from cells, and heightened MMP activity causes an increase in HB-EGF shedding (reviewed in [101]). EGF and cytokines are involved in the regulation of syndecan shedding [21] and KGF induces strong syndecan-1 expression beneath the basement membrane [102]. Further, syndecan-1 expression is strongly upregulated in migrating and proliferating keratinocytes. Syndecan-1 and -4 ectodomains are found in acute dermal wound fluids, where they regulate GF activity [103], specifically the formation of HS-KGF complexes and actions of MMPs on shedding of EGFR ligands [104]. EGFR expression transiently increases after wounding [105] and KGFR is upregulated at the wound margin [106]. Alpha-6 beta-4 integrin, the classic core component of hemidesmosomes, performs adhesive functions by binding to laminin 332 in the basement membrane. Association of EGFR with alpha-6 beta-4 integrin and EGF-induced phosphorylation of beta-4 integrin is important for this disassembly of hemidesmosomes to promote cytokinesis and epithelial migration a wound-healing response (reviewed in [90]). Taken together, our work illustrates additional means by which HPV has adapted to utilize the environment created during wounding, which not only allows the virus access to mitotically active basal cells, but also provides factors essential for the virus to infect cells with the boost of mitogenic signals.

In a broader sense, it is of particular interest to reiterate that syndecans and other HSPG are bound by pathogens in addition to HPV, including some retroviruses, herpesviruses, flaviviruses, and bacteria like *Chlamydia* and *Neisseria* in their infection courses. Some of these pathogens, as discussed above, are also known to activate GFR pathways for infection. This brings up an exciting possibility that these other pathogens might also employ a soluble virus-HS-GF mode of infection under certain circumstances. Our study provides new insights into the transmission of a significant viral pathogen and reveals novel means whereby pathogens may repurpose normal cell functions during infection of their hosts. Likewise, this work uncovers new targets for prophylaxis of HPV, and potentially other pathogen infections.

## Materials and Methods

### Cell culture, transfections, virus production, infections

The sources of different cell lines and their culture conditions, plasmids used, procedures to produce and purify HPV PsV, and the procedure for the exposure and infection of target cells are provided as Supporting Protocols S1 and S2 in Text S1. 293T cells, HaCaT cells, CHO-K1 cells and derivative pgsd-677 were maintained as reported [46], [83], [107], [108]. HPV PsVs encapsidating a luciferase reporter plasmid were generated *via* transfection in 293T cells and quantified for vge and L1/L2 capsid levels. SDS-PAGE and Coomassie Brilliant Blue staining were used to assess the purity of virus stocks [92]. Under our transfection conditions, capsids typically outnumber vge by 2- to 10-fold [88]. CsCl gradient-purified PsV stocks were sonicated, added to cells in various media and incubated at 4°C for 1 h to permit viral attachment. Inocula were aspirated, cells were extensively washed, and fresh culture media or Tyrode's buffer (10 mM HEPES pH 7.4, 130 mM NaCl, 5 mM KCl, 1.4 mM CaCl_2_, 1 mM MgCl_2_, 5.6 mM glucose, and 0.05% BSA) were added. Infections were allowed to proceed at 37°C, typically for 24 h before luciferase quantification. For the co-culture viral release assay, subconfluent donor cells grown on cover slips were incubated with PsVs at ∼2000 vge/cell for 1 h, 4°C (Figure 4A). Cells were washed 3X to remove unbound PsVs, and coverslips transferred to 74-µm mesh plate inserts (Corning). The PsV-exposed donor cell inserts were suspended above a subconfluent recipient cell monolayer with media covering both cultures (Figure 4C). Donor and recipient cell infections were measured by luciferase assay. siRNA cell transfection was performed using Lipofectamine 2000 reagent (Invitrogen), with EGFR siRNA (Cell Signaling) according to manufacturer's recommendations. A nonspecific siRNA was used as a negative control (Dharmacon). Transfection was monitored using fluorescein-conjugated siRNA (Cell Signaling). Scepter Cell Counting (Millipore) for viability and size and Trypan Blue exclusion staining were used to measure cell viability.

### Sepharose 4B gel chromatography and analysis of HMW complexes

Cells were incubated with 200 vge/cell of PsV for 1 h at 4°C, washed 3X with media and incubated at 37°C for various times. Experimental media were cleared by low speed centrifugation and the supernatant was concentrated by Amicon Ultra 30K filtration (Millipore). Concentrated samples were fractionated on Sepharose 4B columns that had been preliminary calibrated with standard proteins as described [34]. The samples were applied to an equilibrated Sepharose 4B column and left for 3 min; eluate was collected as fraction 1. PBS was applied and fraction 2 collected in 3 min and so on. Eluted fractions were analyzed by SDS-PAGE followed by immunoblotting for HPV16 L1, proteoglycans and growth factors. Additional details of chromatography are given in Supporting Protocol S3 in Text S1.

### Gelatin zymography

HaCaT cells were incubated overnight following exposure to HPV PsV (100 vge/cell), culture supernatant was removed, cleared by centrifugation and concentrated by Amicon filtration. Concentrate was mixed with 6× non-reducing sample buffer and electrophoresed through a 8% acrylamide gelatin gel and analyzed as reported [35].

### Fluorescent staining and microscopy

HaCaT cells were seeded onto glass cover slips and cultured overnight. Media were removed and the cells were starved for 2 h with Tyrode's buffer prior to PsV exposure at 4°C, 45 min. AF488-conjugated EGF was added and incubated an additional 15 min. Unbound materials were washed out and cells fixed. After extensive washes, cells were blocked and incubated with rabbit anti-HPV16 VLP antisera. Following PBS washes, slides were incubated with AF594-conjugated anti-rabbit IgG. Alternatively, for visualization of KGFR and HPV co-localization, BSA-blocked cells were incubated with anti-KGFR (FGFR2IIIb) mouse monoclonal and a rabbit anti-HPV VLP antisera. PBS washed slides were incubated with donkey anti-mouse-AF549 and AF488-conjugated anti-rabbit IgG secondary antibodies. For detection of ERK1/2, fixed cells were permeabilized prior to adding anti phospho-44/42 MAPK rabbit monoclonal followed by Cy3-goat anti-rabbit IgG. All images were acquired with a Zeiss LSM 510 META confocal system using appropriate filters. Detailed immunofluorescence methods and antibody specifics are given in Supporting Protocol S4 in Text S1.

### IP and depletions

For co-IP of syndecan-1 from released material, HaCaT cells were seeded and incubated with virus as in Figure 4A with anti-HPV16 L1 mouse mAb attached to Dynabeads–Protein A in the lower chamber (instead of recipient cells as in Figure 4C). After 2 or 20 h of incubation, beads were collected, washed, and solubilized in non-reducing sample buffer. Syndecan-1 was detected using rabbit anti-serum after SDS-PAGE by immunoblot (details in Supporting Protocol S5 in Text S1).

GFRs were subject to co-IP with HPV16 PsVs bound to HaCaT cells at 500 vge/cell at 4°C,1 h; mock-exposed cells were a negative control. Cells were solubilized with cold Triton lysis buffer (1% TX100, 50 mM Tris-HCl, pH 7.5, 150 mM NaCl, 1 mM EDTA, 1 mM PMSF, 10 ng/ml leupeptin, 10 ng/ml aprotinin). Insoluble materials were removed by centrifugation and supernatants were immunoprecipitated for 1 h at 4°C with rabbit anti-HPV16 VLP antibody attached to protein A-magnetic Dynabeads (Invitrogen Dynal). Soluble proteins were resolved by 10% SDS-PAGE and were transferred onto PVDF membranes, which were probed with anti-syndecan-1, anti-HB-EGF, anti-EGF, anti-EGFR, or p-FGFR and then HRP-conjugated secondary Ab. To deplete syndecan-1 and EGF from media, CM was incubated with anti-syndecan-1 mAb or anti-EGF mAb attached to Protein G Sepharose beads for 3 h at RT. The media was filtered to remove the bound material and used for infections. As a negative control we used CM incubated with Protein G Sepharose beads. Additional details of IPs are given in the Supporting Protocol S6 in Text S1.

### EGFR and KGFR signal activation

Subconfluent HaCaT cells were serum-starved for 3–4 h in Tyrode's buffer containing 0.05% BSA. After adding ∼100 vge/cell HPV16 PsVs, 10 ng/ml EGF or 10 ng/ml KFG, cells were incubated at 37°C for 10 min before transferring to ice and solubilizing cells with RIPA buffer. In some experiments cells were incubated with various inhibitors in Tyrode's buffer for 45 min and after Tyrode's washes, were incubated with virus as above in the presence of inhibitors. Lysates were clarified, mixed with Laemmli buffer and boiled for 5 min prior to SDS-PAGE. Immunoblot was performed with various monoclonal and polyclonal antibodies: p-EGFR, p-KGFR, p-ERK, actin.

For nuclear extractions, HaCaT cells were starved 4 h in Tyrode's solution containing 0.05% BSA, then exposed to HPV, EGF or KGF for various times. Cells were solubilized with NP40 lysis buffer and centrifuged. The pellet was incubated with nuclear extraction buffer. Following incubation on ice for 1 h, the extract was clarified and the supernatant subjected to SDS-PAGE and immunoblot for analysis of p-ERK content. Additional details and buffer constituents are given in Supporting Protocol S5 in Text S1.

### Effect of inhibitors on HPV infection

Subconfluent HaCaT cells were pre-treated 45–60 min with 1 µM AG1478 (Calbiochem), 100 nM PD168393 (Calbiochem), 100 µM genistein (Sigma), 100 µM daidzein (Sigma), 1 µM PD173074 (Calbiochem), 100–600 nM cetuximab (ImClone), 1 µM to 100 µM MM (Tocris Bioscience) and 1 µM to 100 µM BM (Tocris Bioscience). For dual inhibitor assays, cells were pre-treated 1 hr with 100 µM monastrol, pre-treated with monastrol plus 500 nM PD168393 for 1 h, or pre-treated with 500 nM PD168393 for 1 hr prior of adding 100 µM monastrol and incubated an additional 1 h. Cells were exposed to HPV16 or HPV31 PsV at 100 vge/cell for 1 h at 4°C, then shifted to 37°C in the presence of inhibitors for 24 h at which time they were analyzed for luciferase expression. These inhibitor concentrations are well documented not to cause cell toxicity; cell viability was ≥94% in each assay.

## Supporting Information

Figure S1 **HPV16 and HPV31 interact with HSPG and syndecan-1 at the cell plasma membrane.** (A–D) Immunofluorescent confocal localization and 3D reconstruction showing HPV16 or HPV31 (green) with heparan sulfate or syndecan-1 (red). PsV were added to cells at 5000 particles per cell. The bars measure 5 µm. (E) IP of HPV16 from HaCaT cells exposed to HPV16 PsV and immunoblot for syndecan-1 (mAb DL-101; Santa Cruz). The bound PsVs and cells were either untreated or membrane-bound proteins were cross-linked with DTSSP. Lane 1, magnetic beads and anti-HPV16; lane 2, IP of HPV16 from HaCaT cells; lane 3, IP of HPV16 from HaCaT cells treated with DTSSP before lysis; lane 4, left blank; lane 5, HaCaT cell lysate (no IP).
(TIF)

Figure S2 **Analysis of Sepharose 4B chromatography of released materials in CM of HaCaT cells exposed to HPV16 PsV.** Eluted fractions (indicated at top of gels) were solubilized in 6× sample buffer, boiled for 3 min. Samples were separated by 10% SDS-PAGE followed by electrotransfer to PVDF membrane. Lanes are indicated below each blot. Membranes were probed for (A) HPV16 L1 using mouse mAb (Abcam) and (B) for syndecan-1 using a monoclonal antibody (Santa Cruz). Lane 11 in Panel B contains HaCaT cell lysate as a control.
(TIF)

Figure S3 **IC_50_ of HPV16 infectivity inhibition by MMP inhibitors batimastat and marimastat.** HaCaT cells incubated with serial dilutions of batimastat (BM) or marimastat (MM) in CM for 1 h before incubation with 100 vge/cell HPV16, 1 h at 4°C. After washing away unbound virus, cells were incubated for 24 h at 37°C in the presence of inhibitors. HPV16 infection was measured with luciferase assay. Error bars represent SEM of three replicates.
(TIF)

Figure S4 **Cell cycle changes induced by inhibitors cannot account for observed HPV16 infection inhibition.** Pyeon *et al.* showed that progression through early M phase is needed for HPV infection of HKs [56]. They also showed that monastrol, which blocks in early M phase leads to an increase in infection (as in Figure 7D). To investigate if infection inhibition by the various agents used in Figure 7 could be attributed to cell cycle changes, identical conditions and timing of inhibitor treatment on HaCaT cells were assayed with propidium iodide and examined using flow cytometry. The fractions of cells in G1 (1n), S (intermediate), and G2/M (2n) phases were expressed as percentages of the total cells counted.
(TIF)

Text S1 **Supporting protocols.**
(DOC)
