# Supplementary figures and images for: Essential Roles for Soluble Virion-Associated Heparan Sulfonated Proteoglycans and Growth Factors in Human Papillomavirus Infections
Source: PLoS Pathog. 2012 Feb 9;8(2):e1002519. doi: 10.1371/journal.ppat.1002519 (PMC3276557; doi:10.1371/journal.ppat.1002519)

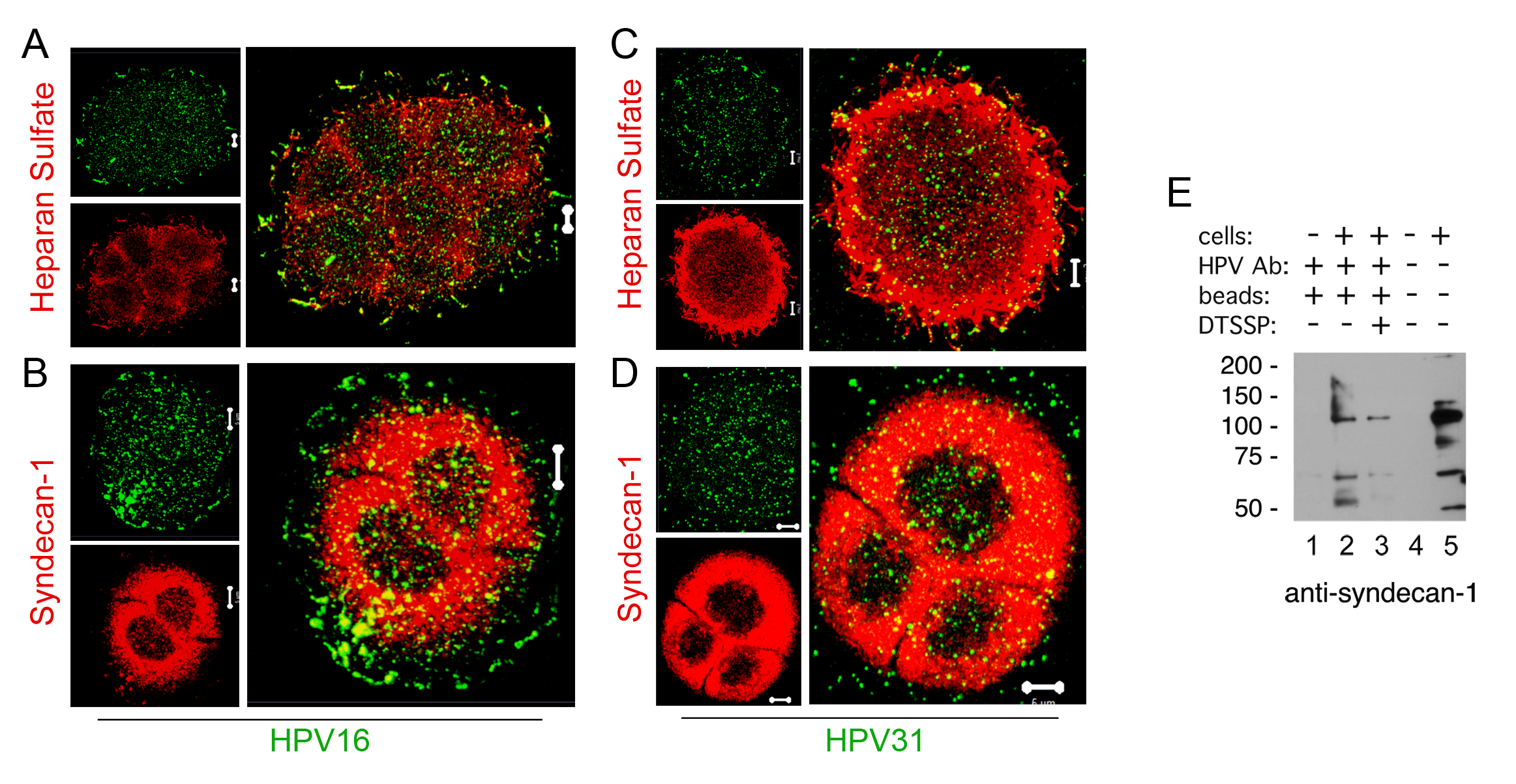

Supplement: Figure S1 — HPV16 and HPV31 interact with HSPG and syndecan-1 at the cell plasma membrane. (A–D) Immunofluorescent confocal localization and 3D reconstruction showing HPV16 or HPV31 (green) with heparan sulfate or syndecan-1 (red). PsV were added to cells at 5000 particles per cell. The bars measure 5 µm. (E) IP of HPV16 from HaCaT cells exposed to HPV16 PsV and immunoblot for syndecan-1 (mAb DL-101; Santa Cruz). The bound PsVs and cells were either untreated or membrane-bound proteins were cross-linked with DTSSP. Lane 1, magnetic beads and anti-HPV16; lane 2, IP of HPV16 from HaCaT cells; lane 3, IP of HPV16 from HaCaT cells treated with DTSSP before lysis; lane 4, left blank; lane 5, HaCaT cell lysate (no IP). (TIF) [file ppat.1002519.s001.tif]

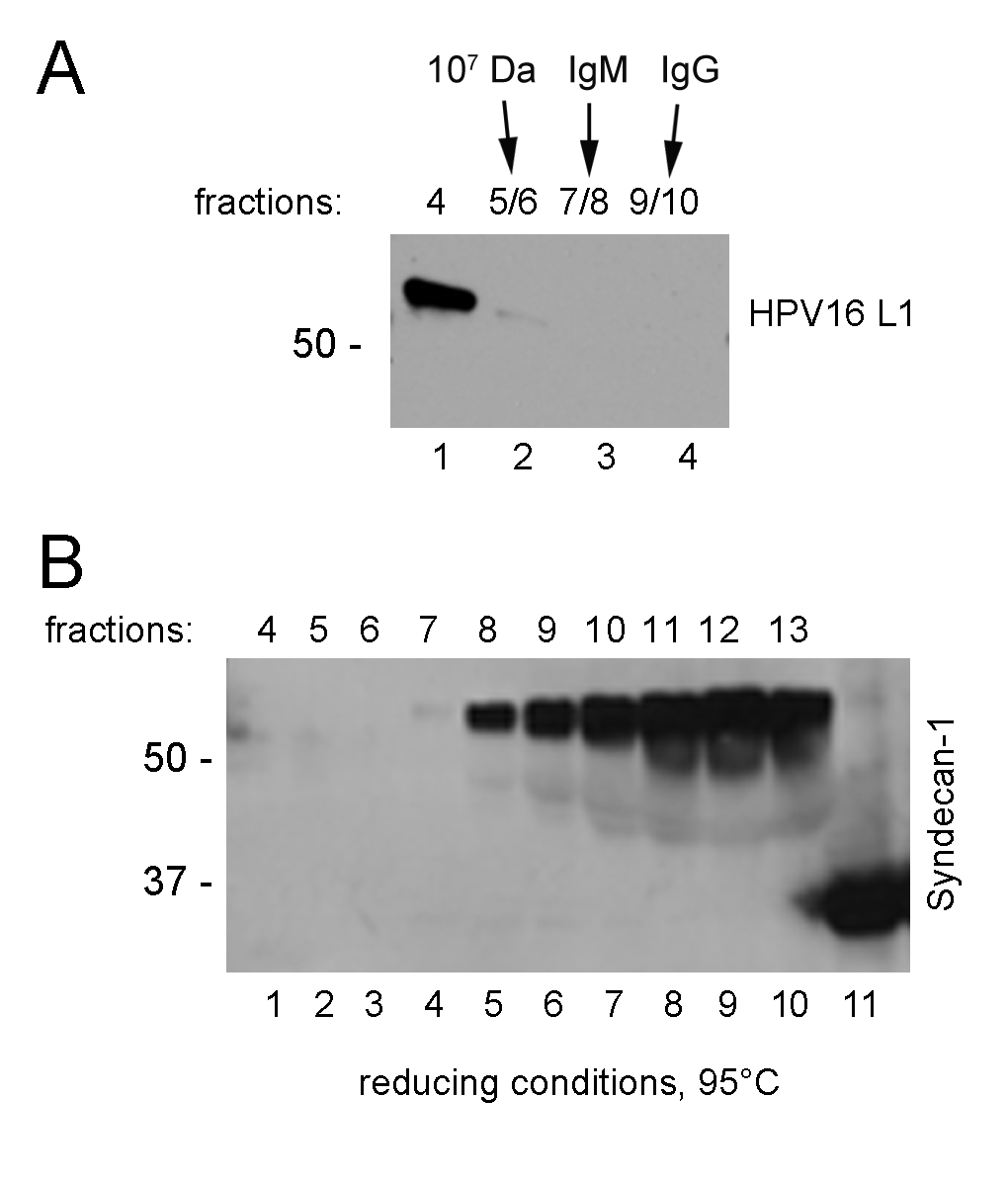

Supplement: Figure S2 — Analysis of Sepharose 4B chromatography of released materials in CM of HaCaT cells exposed to HPV16 PsV. Eluted fractions (indicated at top of gels) were solubilized in 6× sample buffer, boiled for 3 min. Samples were separated by 10% SDS-PAGE followed by electrotransfer to PVDF membrane. Lanes are indicated below each blot. Membranes were probed for (A) HPV16 L1 using mouse mAb (Abcam) and (B) for syndecan-1 using a monoclonal antibody (Santa Cruz). Lane 11 in Panel B contains HaCaT cell lysate as a control. (TIF) [file ppat.1002519.s002.tif]

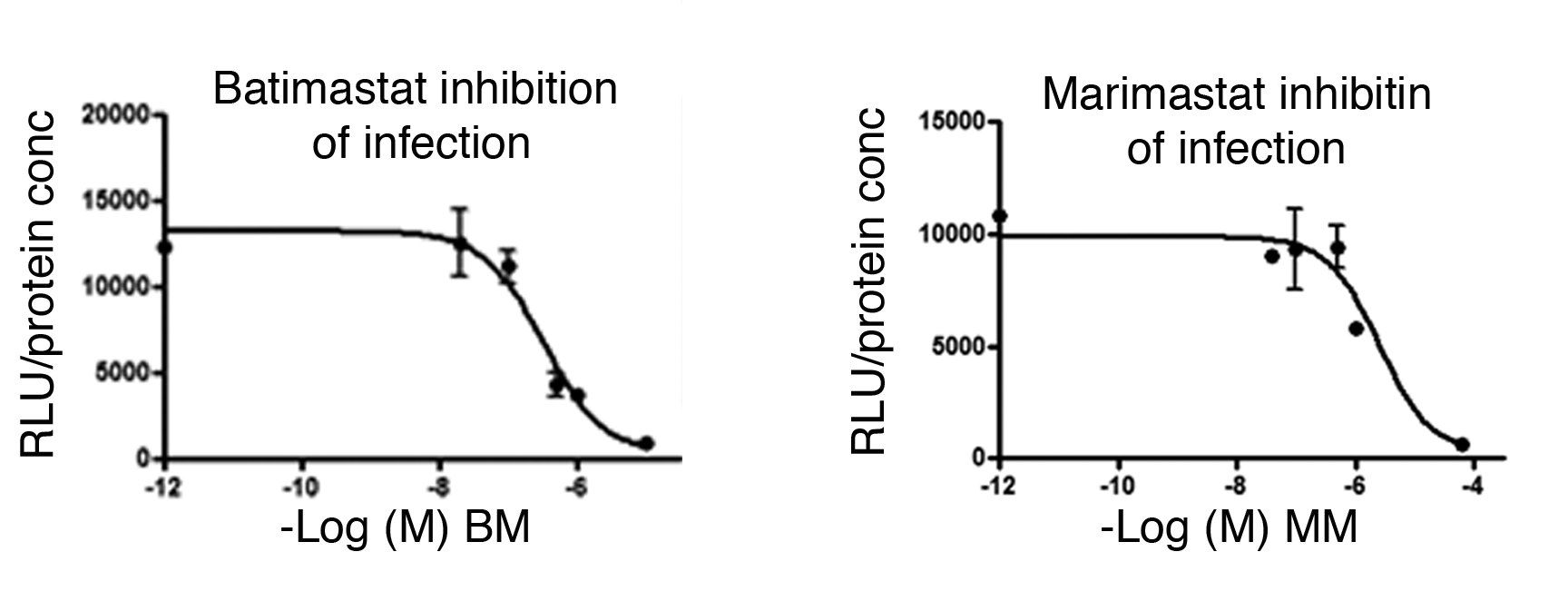

Supplement: Figure S3 — IC50 of HPV16 infectivity inhibition by MMP inhibitors batimastat and marimastat. HaCaT cells incubated with serial dilutions of batimastat (BM) or marimastat (MM) in CM for 1 h before incubation with 100 vge/cell HPV16, 1 h at 4°C. After washing away unbound virus, cells were incubated for 24 h at 37°C in the presence of inhibitors. HPV16 infection was measured with luciferase assay. Error bars represent SEM of three replicates. (TIF) [file ppat.1002519.s003.tif]

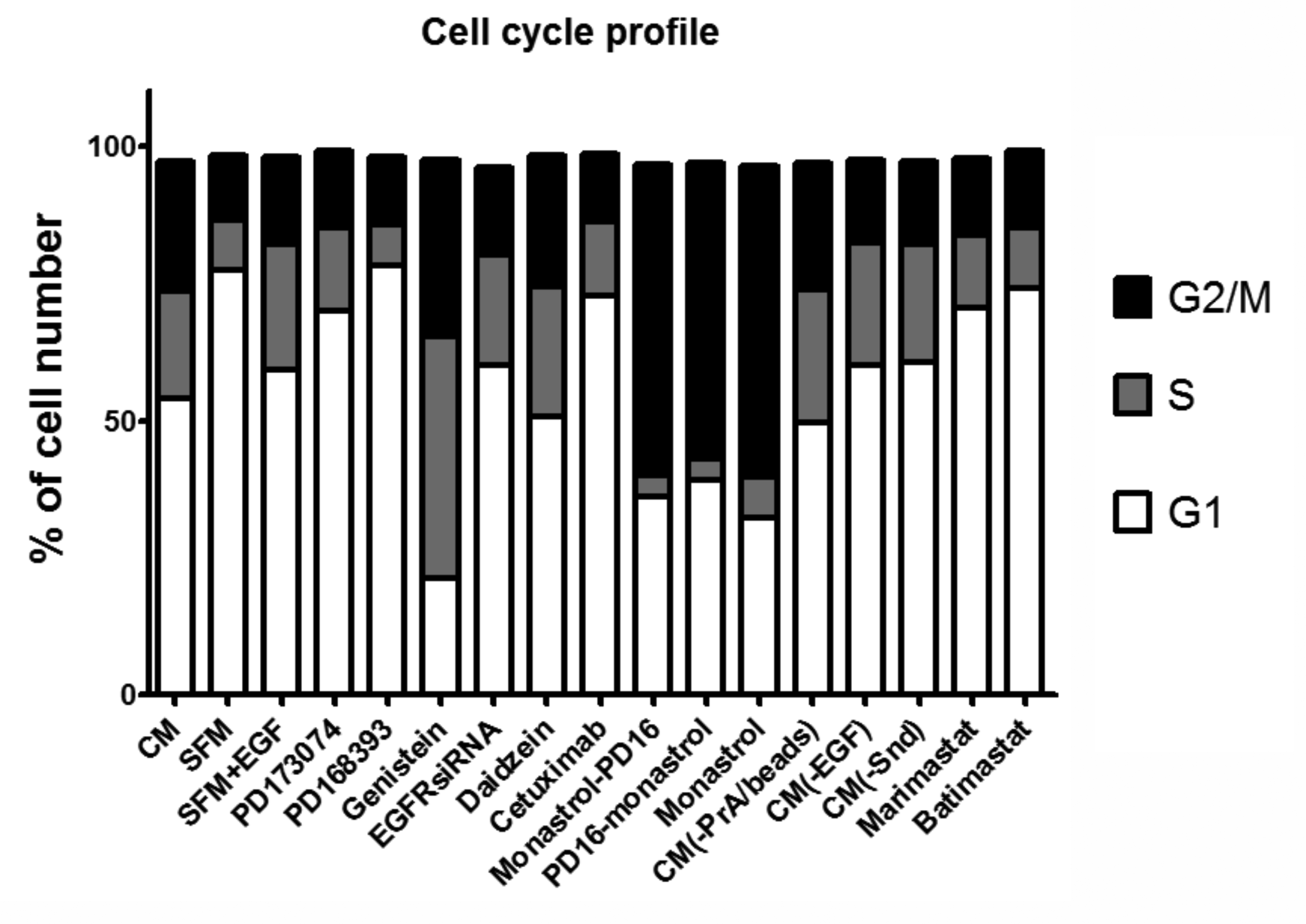

Supplement: Figure S4 — Cell cycle changes induced by inhibitors cannot account for observed HPV16 infection inhibition. Pyeon et al. showed that progression through early M phase is needed for HPV infection of HKs [56]. They also showed that monastrol, which blocks in early M phase leads to an increase in infection (as in Figure 7D). To investigate if infection inhibition by the various agents used in Figure 7 could be attributed to cell cycle changes, identical conditions and timing of inhibitor treatment on HaCaT cells were assayed with propidium iodide and examined using flow cytometry. The fractions of cells in G1 (1n), S (intermediate), and G2/M (2n) phases were expressed as percentages of the total cells counted. (TIF) [file ppat.1002519.s004.tif]
