# Supplementary material for: Essential Roles for Soluble Virion-Associated Heparan Sulfonated Proteoglycans and Growth Factors in Human Papillomavirus Infections
Source: PLoS Pathog. 2012 Feb 9;8(2):e1002519. doi: 10.1371/journal.ppat.1002519 (PMC3276557; doi:10.1371/journal.ppat.1002519)
Supplement: Text S1 — Supporting protocols. (DOC) [file ppat.1002519.s005.doc]

Text S1

SUPPORITNG PROTOCOLS

###### Protocol S1. Cell culture and infections

HaCaT cells are a spontaneously immortalized epithelial line derived from normal adult skin . HaCaT cells were maintained in DMEM/Ham’s F-12 medium (Irvine Scientific), supplemented with 10% FBS (Invitrogen), 4x amino acids (Invitrogen), and glutamine-penicillin-streptomycin (Invitrogen). CHO-K1 cells and their derivative pgsd-677 were grown in Ham's F12 medium supplemented with 10% fetal bovine serum and 1% Glutamax (Invitrogen). HEK-293T cells are derived from a human embryonic kidney cell line immortalized with SV40 large T antigen and were maintained in DMEM high glucose (Irvine Scientific) supplemented with 10% FBS, nystatin/gentamycin (Invitrogen), and 0.4 μg/ml hygromycin B (Roche). HaCaT cells were seeded in to be 60-80% confluent on the day of infection. PsV stocks were sonicated for 30 s, added to cells, and incubated at 4°C for 1h with gentle rocking to permit viral attachment. Inocula were aspirated, cells were washed 3X with complete medium and fresh media were added. Infections were allowed to proceed at 37°C for 20-24h. After washing with PBS, cells were lysed with Promega Luciferase lysis buffer for 10 min at RT. The extracts were centrifuged 0.5 min at 14,000xg, and luciferase activities were measured by using the Luciferase kit assay (Promega) and a Lumat LB 9501 luminometer (Berthold Technologies). Raw luciferase data were normalized by protein content using Bio-Rad protein assay. Control infections were set to 100% infection with the averages of 3-4 replicate experiments and error bars represent standard error of the mean.

**Protocol S2. HPV pseudovirion (PsV) production and purification**

HPV PsVs were generated in 293T cells as described . The transfection-based method for papillomavirus production was modified from that previously published . 293T cells were transfected by the calcium phosphate method with a luciferase reporter (pGL3-control, Promega) and either a codon-optimized HPV31-L1/L2-expressing plasmid or a codon-optimized HPV16-L1/L2-expressing plasmid, pXULL . At 48 h post-transfection, cells were released with trypsin, pelleted, and resuspended at 1x10^8^ cells/ml in Dulbecco’s phosphate-buffered saline (PBS)–9.5 mM MgCl2. Cells were lysed with 0.35% Brij58 and subjected to three freeze-thaw cycles. Unpackaged DNA was digested with 20 U/ml exonuclease V (Epicentre Plasmid-Safe) and 0.3% Benzonase (Sigma). Lysates were allowed to mature overnight and then clarified by low-speed centrifugation. Supernatants were layered atop a 1.25-g/ml to 1.4-g/ml step CsCl gradient. Following centrifugation at 20,000*x*g for 16 to 18 h, the viral band was extracted by side puncture. Virions were washed and concentrated in HSB (25 mM HEPES pH 7.5, 0.5 M NaCl, 1 mM MgCl_2_) using Amicon Ultra-4 100,000 MWCO centrifugation filter units (Millipore). SDS-PAGE and Coomassie Brilliant Blue staining were used to determine virion stock purity and L1 protein content. “Viral genome equivalent” (vge) titers of packaged reporter plasmids were determined by dot blot hybridization or qPCR as previously described .

**Protocol S3. Sepharose 4B gel chromatography and analysis of HMW complexes**

Sepharose 4B columns were preliminary calibrated with standard proteins as described . HaCaT cells were incubated with 200 vge/cell of HPV PsVs for 1h at 4°C, washed 3X with media and incubated at 37°C for various times before harvesting the experimental media. Media were subjected to low speed centrifugation to remove debris and the supernatant was concentrated on by Amicon Ultra 30K filtration (Millipore). Concentrated samples were fractionated on a 1 ml Sepharose 4B column that had been washed with PBS; 0.1 ml of the sample was applied at the top and left to enter the gel for 3 min; the 0.1 ml of the eluate was collected as fraction 1. Next, 0.1 ml of PBS was applied, and fraction 2 collected in 3 min and so on. The void volume fraction of this gel (fraction 4) contains large complexes or particles (>10^7^ Da); maxima of MW standards IgM and IgG elute in fractions 8 and 10, respectively . The mini-columns were used in order to minimize the time necessary for separation; in preliminary experiments it was found that the quality of separation was comparable to that obtained with 25 ml columns. The eluted fractions were analyzed by SDS-PAGE followed by immunoblotting for HPV16 L1, proteoglycans and growth factors. Primary antibodies included monoclonal anti-HPV16 L1 (Abcam), anti HB-EGF (Santa Cruz), anti-amphiregulin (Santa Cruz), anti-EGF (ProSpec-Tany TechnoGene) and anti-heparan sulfate (Millipore), rabbit serum to syndecan-1 (anti-Snd; Millipore).

**Protocol S4. Immunofluorescence staining and microscopy**

HaCaT cells were seeded onto glass cover slips in a 12-well plate and cultured overnight. The media were removed, and the slides were incubated with Tyrode’s buffer. After 2 h of starvation, cells were incubated with HPV PsVs at 4°C. After 45 min of incubation in wells Alexa 488-EGF (Invitrogen) was added and incubated an additional 15 min. Unbound EGF and virus were washed out with cold Tyrode’s solution and cells fixed with 4% paraformaldehyde for 45 min at RT. After several washes with PBS, cells were blocked with 1% BSA containing Tyrode’s solution for 1 h at RT, and incubated with rabbit polyclonal antibody (1:200) against HPV16 or HPV31 for 1 h at RT (antibody raised to pure VLPs made in our lab, and affinity purified on Protein A column). Following several washes with PBS, slides were incubated with DyLight^TM^ 594-conjugated affinityPure donkey anti-rabbit IgG (1:200; Jackson Immunochem.) for 45 min at RT. Cells were washed again with PBS (5 times, 10 min each) and coverslips were inverted onto Prolong Gold mounting solution. Alternatively, for visualization of KGFR and HPV co-localization, BSA-blocked cells were incubated with Bek (C8) mouse mAb (Santa Cruz Biotech.) and rabbit polyclonal anti-HPV16 or anti-HPV31 VLP for 1 h at RT (primary antibodies diluted 1:100 in 1% BSA/Tyrode’s buffer). After washing with PBS, slides were incubated with donkey anti-mouse-AF549 and donkey anti-rabbit-AF488 IgG secondary antibodies (1:200 dilution in 1% BSA/Tyrode’s; both Jackson Immunochemicals). For detection of intracellular p-ERK1/2, fixed cells were permeabilized with Tyrode’s buffer containing 0.1% Triton-X100 for 5 min. Anti-p-44/42 MAPK rabbit mAb (1:200; Cell Signaling) was used as primary antibody and goat anti rabbit Cy3 (Jackson Immunochem.) was used as a secondary antibody. Vectashield mounting medium with DAPI (H-1200; Vector Laboratories, Inc.) was used.

For detection of HPV and syndecan HSPG localization, HaCaT cells seeded on coverslips were exposed to 5000 HPV PsVs per cell for 1h at 4°C. Cells were fixed with 4% paraformaldehyde for 40min and permeabilized with 0.1% Triton-X 100 for 5min. Cells were blocked with 1% BSA containing Tyrode’s solution for 1 h at RT, and incubated with primary antibodies (Rabbit polyclonal antibody of HPV16 or HPV31, and mouse monoclonal antibodies against syndecan-1 (Santa Cruz) or HS (Millipore) were used at 1:200 dilution in Tyrode’s buffer containing 1% BSA for 1 h at RT. Following several washes with PBS, slides were incubated with DyLight^TM^ 488-conjugated affinityPure donkey anti-rabbit IgG (1:200; Jackson Immunochem.) and DyLight^TM^ 594-conjugated anti-mouse for 45 min at RT. Cells were washed again with PBS (5 times, 10 min each) and coverslips were inverted onto Prolong Gold mounting solution.

All images were acquired with a Zeiss LSM 510 META confocal system using appropriate filters. Parameters of lasers intensities were kept constant during the imaging. 3D (full projection) cell imaged were generated with Zen 2009 software (Zeiss), using Z-stack confocal series. Fluorescent images in this paper were generated in the UNM Cancer Center Fluorescence Microscopy Facility, supported as detailed on the webpage: <http://hsc.unm.edu/crtc/microscopy/index.html>.

**Protocol S5. SDS-PAGE and immunoblotting**

Subconfluent HaCaT cells were serum-starved for 3-4 h in Tyrode’s buffer supplemented with 0.05% BSA. After adding ~100 vge/cell HPV16 PsV, cells were incubated at 37°C for 10 min before transferring to ice and solubilizing cells with RIPA buffer (50 mM Tris pH 7.5, 0.1% SDS, 1% sodium deoxycholate, 150 mM NaCl, 1 mM EDTA, 1% Triton X-100, 1mM sodium vanadate, 1mM PMSF, 1 mg/ml leupeptin, 1 mg/ml aprotinin and 1 mg/ml pepstatin). In experiments with various inhibitors, cells incubated with indicated of concentration (see figure legends) of inhibitors in Tyrode’s buffer for 45 min and after Tyrode’s buffer washes were incubated with virus as above in the presence of inhibitors. Lysates were centrifuged at 14,000×*g* for 10 min, mixed with 6× Laemmli buffer and heated at 95°C for 5 min. Samples were analyzed by SDS-PAGE, and proteins transferred to PVDF membranes. Membranes were probed with various monoclonal and polyclonal antibodies: p-EGFR (Cell Signaling), p-KGFR (p-FGFR2IIIb) (Cell Signaling), p-ERK (Cell Signaling), actin (MP Biomedical). Horseradish peroxidase-labeled secondary antibodies were used to detect antigen-antibody interactions (Pierce, Amersham Pharmacia Biotech). Nuclear fractionation was performed for detection of p-ERK movement into the nucleus. HaCaT cells were starved for 4h in Tyrode’s solution containing 0.05% BSA. After incubation of cells with HPV16, HPV31 or EGF for various times, cells were solubilized with NP40 lysis buffer (10 mM HEPES pH 7.9, 1.5 mM MgCl_2_, 10 mM KCl, 1 mM PMSF, 1 mM sodium vanadate, 10 mg/ml leupeptin, 10 mg/ml pepstatin, 10 mg/ml aprotinin, 0.1% NP-40). After centrifugation at 4,000×g for 10 min the pellet was incubated with nuclear extraction buffer (20 mM HEPES pH 7.9, 10% glycerol; 1.5 mM MgCl_2_, 400 mM KCl, 1 mM PMSF; 1mM Na_3_VO_4_). Following incubation on ice for 1h, the extract was centrifuged for 20 min at 16,000×g and the supernatant was used as nuclear fraction and subjected to 10% SDS-PAGE and immunoblot for detection of p-ERK1/2 as above.

**Protocol S6. Immunoprecipitations**

Confluent HaCaT cells were seeded as donor cells and incubated with 500 vge/cell HPV16 PsVs at 4°C for 1 h as in Fig. 3A with anti-HPV16 L1 mouse mAb attached to Dynabeads–Protein A in the lower chamber (instead of recipient cells as in Fig. 3C). After 2 or 20 h of incubation, beads were collected, washed several times with PBS, and solubilized in non-reducing sample buffer. Syndecan-1 was detected by immunoblot with anti-Snd-1 rabbit serum (Millipore).

To detect the association of GFR with HPV16, confluent HaCaT cells were incubated with 500 vge/cell HPV16 PsVs at 4°C for 1 h; a second plate at the same confluence was left unexposed to PsVs (for negative control). Plates were then washed twice with cold PBS and cells were solubilized with 2 ml/plate of cold lysis buffer (1% TX100, 50 mM Tris-HCl, pH 7.5, 150 mM NaCl, 1 mM EDTA, 1 mM PMSF, 10 ng/ml leupeptin, 10 ng/ml aprotinin). Insoluble materials were removed by centrifugation at 13,000 rpm for 10 min and supernatants were immunoprecipitated with affinity-purified rabbit polyclonal anti-HPV16 VLP antisera attached to protein A-magnetic Dynabeads (Invitrogen Dynal) for 1 h at 4°C. Beads were washed twice with cold lysis buffer, twice with cold PBS, solubilized in 15 ml Laemmli sample buffer and boiled for 5 min. Soluble proteins were resolved by 10% SDS-PAGE and were electro-blotted onto PVDF membranes. Membranes were probed with anti-syndecan-1 mAb (Santa Cruz), anti-EGFR or p-FGFR antibodies (Cell Signaling). A 1:10,000 dilution of rabbit TrueBlot HRP-conjugated anti-rabbit IgG (eBioscience) in TBS plus 0.05% Tween 20 and 1% BSA was used as a secondary antibody.

To detect PsV interaction with syndecan-1, cells were exposed to PsV at 5000 particles per cell. The cultures were untreated or incubated with 1mM DTSSP (3,3´-dithiobis [sulfosuccinimidyl-propionate]) as a water-soluble cross-linker in cold PBS and incubated for 30 min at 4°C. Cells were washed with cold PBS and quenched with 50 mM Tris pH 7.5 for 15 min at 4°C. After washing twice with cold PBS cells in all plates were solubilized with 2ml/plate of cold lysis buffer (1% TX100, 50 mM Tris-HCl, pH 7.5, 150 mM NaCl, 1 mM EDTA, 1 mM PMSF, 10 ng/ml leupeptin, 10 ng/ml aprotinin). Insoluble materials were removed by centrifugation at 13,000 rpm for 10 min and supernatants were immunoprecipitated by anti HPV16 antibody with protein A-magnetic Dynabeads (Invitrogen Dynal) for 1h at 4°C. Beads washed twice with cold lysis buffer, twice with cold PBS, solubilized in 15ul Laemmli sample buffer and boiled for 5 min. Solubilized proteins were resolved by 10% SDS-PAGE and were electroblotted onto PVDF membranes. Membranes were probed with anti syndecan-1 mAb (Santa Cruz).

To prepare syndecan-1 or EGF depleted medium, 20 μg anti-syndecan-1 mAb (Santa Cruz) or anti-EGF mAb (Santa Cruz) attached to Protein G Sepharose beads (GE healthcare) was washed with PBS and incubated with 3 ml of 10% FCS-DMEM for 3 h at RT. The suspension was filtered with a 0.2-μm filter (Nalgene). 10% FCS-DMEM incubated with Protein G Sepharose beads were used as a negative control.

**REFERENCES**

Boukamp, P., Petrussevska, R.T., Breitkreutz, D., Hornung, J., Markham, A., and Fusenig, N.E. (1988). Normal keratinization in a spontaneously immortalized aneuploid human keratinocyte cell line. J. Cell Biol. *106*, 761-771.

Buck, C.B., Pastrana, D.V., Lowy, D.R., and Schiller, J.T. (2005a). Generation of HPV pseudovirions using transfection and their use in neutralization assays. In Human Papilloma Viruses: Methods and Protocols, C. Davy, and J. Doorbar, eds. (Totowa, NJ, Humana Press, Inc.), pp. 447-464.

Buck, C.B., Thompson, C.D., Pang, Y.-Y.S., Lowy, D.R., and Schiller, J.T. (2005b). Maturation of Papillomavirus Capsids. J. Virol. *79*, 2839-2846.

Buck, C.B., Cheng, N., Thompson, C.D., Lowy, D.R., Steven, A.C., Schiller, J.T., and Trus, B.L. (2008). Arrangement of L2 within the Papillomavirus Capsid. J. Virol. *82*, 5190-5197.

Campos, S.K., and Ozbun, M.A. (2009). Two highly conserved cysteine residues in HPV16 L2 form an intramolecular disulfide bond and are critical for infectivity in human keratinocytes. PLoS ONE *4*, e4463.

Cinek, T., and Horejsi, V. (1992). The nature of large noncovalent complexes containing glycosyl-phosphatidylinositol-anchored membrane glycoproteins and protein tyrosine kinases. J Immunol *149*, 2262-2270.

Otáhal, P., Angelisová, P., Hrdinka, M., Brdicka, T., Novák, P., Drbal, K., and Horejsí, V. (2010). A New Type of Membrane Raft-Like Microdomains and Their Possible Involvement in TCR Signaling. J. Immunol. *184*, 3689-3696.

Ozbun, M.A. (2002). Infectious human papillomavirus type 31b: purification and infection of an immortalized human keratinocyte cell line. J Gen Virol *83*, 2753-2763.

Patterson, N.A., Smith, J.L., and Ozbun, M.A. (2005). Human papillomavirus type 31b infection of human keratinocytes does not require heparan sulfate. J Virol *79*, 6838-6847.

Smith, J.L., Campos, S.K., and Ozbun, M.A. (2007). Human papillomavirus type 31 uses a caveolin 1- and dynamin 2-mediated entry pathway for infection of human keratinocytes. J Virol *81*, 9922-9931.
